# Identification of *SPP1* ^+^ macrophages as an immune suppressor in hepatocellular carcinoma using single-cell and bulk transcriptomics

**DOI:** 10.3389/fimmu.2024.1446453

**Published:** 2024-12-03

**Authors:** Han Jin, Woonghee Kim, Meng Yuan, Xiangyu Li, Hong Yang, Mengzhen Li, Mengnan Shi, Hasan Turkez, Mathias Uhlen, Cheng Zhang, Adil Mardinoglu

**Affiliations:** ^1^ Central Laboratory, Tianjin Medical University General Hospital, Tianjin, China; ^2^ Science for Life Laboratory, KTH – Royal Institute of Technology, Stockholm, Sweden; ^3^ Department of Medical Biology, Faculty of Medicine, Atatürk University, Erzurum, Türkiye; ^4^ Centre for Host-Microbiome Interactions, Faculty of Dentistry, Oral & Craniofacial Sciences, King’s College London, London, United Kingdom

**Keywords:** hepatocellular carcinoma, co-expression network, tumor-associated macrophage, macrophage heterogeneity, single-cell sequencing

## Abstract

**Introduction:**

Macrophages and T cells play crucial roles in liver physiology, but their functional diversity in hepatocellular carcinoma (HCC) remains largely unknown.

**Methods:**

Two bulk RNA-sequencing (RNA-seq) cohorts for HCC were analyzed using gene co-expression network analysis. Key gene modules and networks were mapped to single-cell RNA-sequencing (scRNA-seq) data of HCC. Cell type fraction of bulk RNA-seq data was estimated by deconvolution approach using single-cell RNA-sequencing data as a reference. Survival analysis was carried out to estimate the prognosis of different immune cell types in bulk RNA-seq cohorts. Cell-cell interaction analysis was performed to identify potential links between immune cell types in HCC.

**Results:**

In this study, we analyzed RNA-seq data from two large-scale HCC cohorts, revealing a major and consensus gene co-expression cluster with significant implications for immunosuppression. Notably, these genes exhibited higher enrichment in liver macrophages than T cells, as confirmed by scRNA-seq data from HCC patients. Integrative analysis of bulk and single-cell RNA-seq data pinpointed *SPP1* ^+^ macrophages as an unfavorable cell type, while *VCAN* ^+^ macrophages, *C1QA* ^+^ macrophages, and *CD8* ^+^ T cells were associated with a more favorable prognosis for HCC patients. Subsequent scRNA-seq investigations and *in vitro* experiments elucidated that SPP1, predominantly secreted by *SPP1* ^+^ macrophages, inhibits *CD8* ^+^ T cell proliferation. Finally, targeting SPP1 in tumor-associated macrophages through inhibition led to a shift towards a favorable phenotype.

**Discussion:**

This study underpins the potential of SPP1 as a translational target in immunotherapy for HCC.

## 1 Introduction

The advent of immunotherapy has ushered in a new era for treating various cancers, relying on immune system activation and reprogramming. Strategies like inhibiting immune checkpoints (e.g., PD-1/PD-L1 and CTLA-4) have shown promise in enhancing the immune system’s ability to target and eliminate tumors (1). Liver cancer – a leading cause of global cancer-related mortality (2), presents a unique challenge due to its often asymptomatic nature, making early detection and treatment opportunities scarce (3). Hepatocellular carcinoma (HCC) is the most common type of primary liver cancer. Therefore, novel therapeutic strategies that rely on immunotherapy are of great interest for HCC treatment.

Despite the progress, a significant number of advanced HCC patients do not respond to current immunotherapies, underscoring the need for more effective treatment targets and combination strategies (4). This necessitates a deeper understanding of the tumor immune microenvironment (TIME), particularly the functional diversity of liver macrophages and T cells, and their intricate interactions within the TIME – a domain still partially explored (5). Substantial efforts and an abundance of omics data, facilitated by the widespread use of bulk and single-cell RNA-seq (scRNA-seq) transcriptomics data, have sought to unravel the intricacies of the liver TIME (6–9). Notably, scRNA-seq studies, providing gene expression profiles at a single-cell level (10), have significantly expanded our understanding of immune cell type heterogeneity in HCC and other cancers (11). However, due to the high costs associated with scRNA-seq experiments and computational resources, scRNA-seq cohorts are often constrained by limited sample sizes. This necessitates the translation and validation of scRNA-seq findings on a larger scale, such as using bulk RNA-seq cohorts, especially when these bulk RNA-seq cohorts are well-documented with extensive clinical information. As exemplified by The Cancer Genome Atlas (TCGA) project, such large-scale bulk RNA-seq datasets offer an opportunity to correlate scRNA-seq discoveries with patients’ survival outcomes for robust results (12).

This study addresses these challenges by concurrently analyzing the bulk and single-cell RNA-seq transcriptomics data to estimate immune cell heterogeneity and its impact on HCC patients’ survival. We employed gene co-expression network and regulatory analysis using bulk RNA-seq data from two independent HCC cohorts: the TCGA Liver Hepatocellular Carcinoma (TCGA-LIHC or LIHC) and the Japanese Liver Cancer – Riken cohort from the International Cancer Genome Consortium (ICGC-LIRI-JP or LIRI). These analyses aimed to identify consensus biological insights. To associate these findings with specific liver cell types, we integrated a scRNA-seq dataset (GSE166635) for HCC (13). Subsequently, we used the scRNA-seq data as a reference to estimate the relative proportions of heterogeneous immune cell types within the HCC tumors and associate their contributions to patient survival. Additionally, we analyzed interactions among the key immune cells and evaluated the effects of inhibition of the key gene, *SPP1* in tumor-associated macrophages.

## 2 Materials and methods

### 2.1 Bulk RNA sequencing data acquisition and processing

Two independent RNA-seq data TCGA-LIHC (14) and ICGC-LIRI-JP (15) for hepatocellular carcinoma were selected for the network-based analysis. For TCGA-LIHC, read counts were downloaded from the GDC Data Portal (https://portal.gdc.cancer.gov/). For ICGC-LIRI-JP, read counts were downloaded from the ICGC Data Portal (https://dcc.icgc.org/). For both cohorts, primary tumors and adjacent normal tissues were included, resulting in a total of 421 samples obtained from the LIHC (371 tumors and 50 normal samples), and 437 samples (240 tumors and 197 normal samples) obtained from the LIRI cohort.

The count data of the two cohorts were processed in the same way. First, only protein-coding genes obtained by the R package biomaRt (v2.50.1) (16) were included in the downstream analysis. Then, lowly expressed genes that have a count of less than 10 in more than 90% of the samples were filtered out. The count data were subsequently normalized and transformed by variance stabilizing transformation (VST) using the R package DESeq2 (v1.34.0) (17). As genes with a low variance do not contribute to the clustering analysis but introduce noise, after examining the histograms of gene variance based on the VST expression (data not shown), the top 15,000 most variable genes were selected for downstream analyses. Gene identifiers were kept as original (Ensembl ID for TCGA-LIHC; HUGO Gene Nomenclature Committee HGNC gene symbol for ICGC-LIRI-JP) and were converted by biomaRt when needed.

The publicly available data GSE230666 (18) was used to investigate the inhibitory effect of *SPP1* in HCC-TAMs. In this study, THP-1 cells were differentiated into M0 macrophages by 24 h incubation with 150 mM phorbol-12-myristate 13-acetate (PMA) and were subsequently cultured with the supernatant of HepG2 liver cancer cells after starvation treatment to develop HCC-TAMs. *SPP1* shRNA was induced to inhibit the expression of *SPP1* in HCC-TAMs (sh*SPP1*), with shControl generated for comparison. Each condition has two samples. Transcript expression was quantified from the fastq files using kallisto (v0.48.0) (19) based on the Ensembl Homo sapiens reference cDNA. The transcript expression was assembled to the gene level using the R package tximport (v1.28.0) (20), with only protein-coding transcripts and genes included. The lowly expressed genes with an average count below 5 were filtered out, resulting in 13,821 genes for downstream analysis.

### 2.2 Gene co-expression network analysis

Weighted gene co-expression network analysis (WGCNA, v1.70) (21) was used to construct gene co-expression networks (GCNs) for LIHC and LIRI cohorts, respectively, with both tumor and normal samples included, to identify co-expressed genes preserved in both phenotypes. The GCNs were constructed based on an adjacency matrix of signed correlations between gene pairs:


$$a_{i,j}={\left[\frac{cor\left(gene_{i},gene_{j}\right)+1}{2}\right]}^{\beta }$$

where $a_{i,j}$ is the signed correlation between gene i and j , cor is the Pearson’s Correlation Coefficient (PCC), and β is the soft-thresholding power value to force the adjacency matrix to fit a scale-free topology. In this way, the correlation between gene pairs measured by PCC was scaled to lie between 0 (not connected) and 1 (fully connected), where 0 equals the value -1 of PCC, and 1 equals the value 1 of PCC. Under the soft-thresholding power values of 12 for LIHC and 14 for LIRI, both networks achieved the scale-free topology criterion (22), with a scale-free fitting index of 0.854 for LIHC and 0.861 for LIRI. Hierarchical clustering was made based on the dissimilarity of the topological overlap matrix (TOM) (21) created from the adjacency matrix. This resulted in 22 and 23 gene co-expression clusters constructed from LIHC and LIRI cohorts, denoted as LIHC-1 to LIHC-22 and LIRI-1 to LIRI-23, respectively (**Supplementary Table S2**).

The reproducibility and preservation of the LIHC clusters in the LIRI cohort were tested by the function *modulePreservation()* provided in the WGCNA package. In short, a summarized z-score (Z_summary_) combining multiple cluster preservation statistics (23) was obtained for each LIHC cluster, indicating the level of cluster preservation and reproducibility in the independent LIRI cohort. As suggested, a Z_summary_ > 10 indicates strong cluster preservation; 2 < Z_summary_ < 10 for weak preservation; and Z_summary_ < 2 for no preservation (23).

In addition, we deployed differential co-expression analysis (24) on the LIHC cohort to identify tumor- or normal-specific GCNs. First, two adjacency matrices based on normal tissues and tumor samples, respectively, were established using the following formula:


$$a_{i,j}^{type}=cor\;\left(gene_{i},gene_{j}\right)$$

Then, the differential adjacency matrix was calculated as follows:


$$d_{i,j}{\left(\sqrt{\frac{1}{2}|sign\left(a_{i,j}^{tumor}\right)\times {\left(a_{i,j}^{tumor}\right)}^{2}-sign\left(a_{i,j}^{normal}\right)\times {\left(a_{i,j}^{normal}\right)}^{2}|}\right)}^{\beta }$$

Given the soft-thresholding power value β of 4, the differential adjacency matrix achieved scale-free topology (scale-free fitting index = 0.864). Similar to the conventional WGCNA, hierarchical clustering was applied to the TOM based on the differential adjacency matrix, resulting in 2,121 differentially co-expressed genes between normal and tumor samples, and the genes were clustered into 11 differential co-expression clusters (denoted as LIHC-Diff-1 to LIHC-Diff-11, see **Supplementary Table S2**).

### 2.3 Consensus regulatory network construction

A consensus gene regulatory network (GRN) integrating LIHC and LIRI cohorts for HCC (including normal tissues and tumors) was constructed using the cosifer (v0.0.5), a Python package for consensus inference of GRN by integrating different expression-based regulatory inference algorithms (25). For this, a total of 1,639 human transcription factors (TFs) were used as the candidate TFs (26). As an example, for LIHC expression data, seven meta-GRNs were constructed using GENIE3 (R package GENIE3, v1.16.0) (27), ARACNe-a and ARACNE-m (additive and multiplicative model of ARANCE, Algorithm for the Reconstruction of Gene Regulatory Networks) (28), CLR (Context Likelihood of Relatedness) (29), MRNET (Minimal Redundancy Maximal Relevance based network) (30), Pearson’s Correlation Coefficient, and Spearman’s Correlation Coefficient. Here, the ARACNe-a, ARACNE-e, CLR, and MRNET regulatory networks were established by the R package parmigene (v1.1.0) (31). Then, these GRNs were combined by the Strategy for Unsupervised Multiple Method Aggregation (SUMMA) (32) to get a consensus GRN for LIHC. The SUMMA is an unsupervised ensemble learning algorithm that can estimate the performance of each learning model (i.e., a GRN) and combine different GRNs into a consensus GRN. The whole GRN construction workflow was applied to both the LIHC and LIRI cohorts to obtain a series of meta-GRNs (each cohort = 7). Those meta-GRNs were integrated by the cosifer to obtain a GRN for LIHC, a GRN for LIRI, and finally, a comprehensive GRN integrating all the 14 meta-GRNs from both cohorts.

ChIP-X Enrichment Analysis 3 (ChEA3) (33) was used to identify the potential TFs for the common immune cluster (LIHC-5 ∩ LIRI-11) based on the TF-targets knowledge databases. The analysis was done by the online tool (https://maayanlab.cloud/chea3/) by querying the intersected genes between LIHC-5 and LIRI-11.

### 2.4 Single-cell RNA sequencing data acquisition and processing

Read counts of the HCC single-cell RNA sequencing data GSE166635 (13) were downloaded from the Gene Expression Omnibus (GEO) as the discovery cohort. This cohort includes two primary HCC samples HCC1 (16,077 cells) and HCC2 (9,112 cells) from two patients, respectively. As described in the original publication (13), the data have been processed by CellRanger (v3.1.0) using the GRCh38 as the reference genome. Further processing procedures were performed in R by us, filtering out cells with 1) less than 200 detected genes; 2) less than 5% ribosomal reads; 3) more than 20% mitochondrial reads. We also removed gene *MALAT1*, mitochondrial genes, and genes expressed in less than three cells. In addition, doublets were detected by the R package DoubletFinder (v2.0.3) (34) and were removed. After quality control, the final data have 23,605 genes expressed in 18,724 cells.

Gene expression values of HCC1 and HCC2 were normalized by *NormalizeData()* function, and cells from the two samples were integrated by Seurat (v4.0.5) (35) using an anchor-based approach (36) based on the 2,000 most variable genes in each sample. Then, the integrated data were scaled by *ScaleData()*, and at the same time the mitochondrial and ribosomal contamination, as well as the difference between the G2M cell cycle scores and S phase cell cycle scores calculated by *CellCycleScoring()* function were regressed out. In this way, the signals separating non-cycling and cycling cells were maintained, and the differences between cell cycle phases were removed.

Having reduced the dimensions of the data by principal component analysis (PCA) and examined the statistical significance of each principal component (PC) by the Jack Straw method (data not shown), the cells were clustered by the Louvain algorithm (37) based on the first 40 PCs, with the parameter resolution = 0.5. This resulted in a total of 17 cell clusters including four macrophage subpopulations characterized by the high expression of *CD68*, and four T cell subpopulations characterized by the high expression of *CD3E*. Further clustering analysis was done on the T cells, splitting the whole T cell population into five, thus in total 18 cell clusters were identified. The cell clusters were annotated manually based on the markers previously identified for liver and immune cells (6–8, 10, 38).

In addition, two independent cohorts GSE140228 (8) and GSE156337 (39) for liver macrophages in HCC were selected for validation. For GSE140228, read counts of the droplet-based data (66,187 cells) were downloaded from GEO, whereas the SMART-seq2 data were excluded due to a relatively low number of cells (n = 7,074). Cells annotated as “Mφ-C1-THBS1” (n = 1,005) and “Mφ-C2-C1QA” (n = 1,702) in normal and tumor (core + edge) samples collected from four HCC patients were selected for further analysis. The other liver-derived myeloid cells were excluded due to insufficient cell amounts. Read counts were normalized by the Seurat function *SCTransform()*. Meanwhile, the mitochondrial and ribosomal contamination and the cell cycling difference between the G2M and S phases were regressed out. Cells from different patients were integrated by the R package harmony (v0.1.0) (40). After that, the top 10 macrophage markers identified in the discovery cohorts for *C1QA* ^+^, *SPP1* ^+^, and *VCAN* ^+^ Mφ (see below differential expression analysis) were verified for their expression in the GSE140228 cohort by the Seurat function *AddModuleScore().*

**Figure 1:**
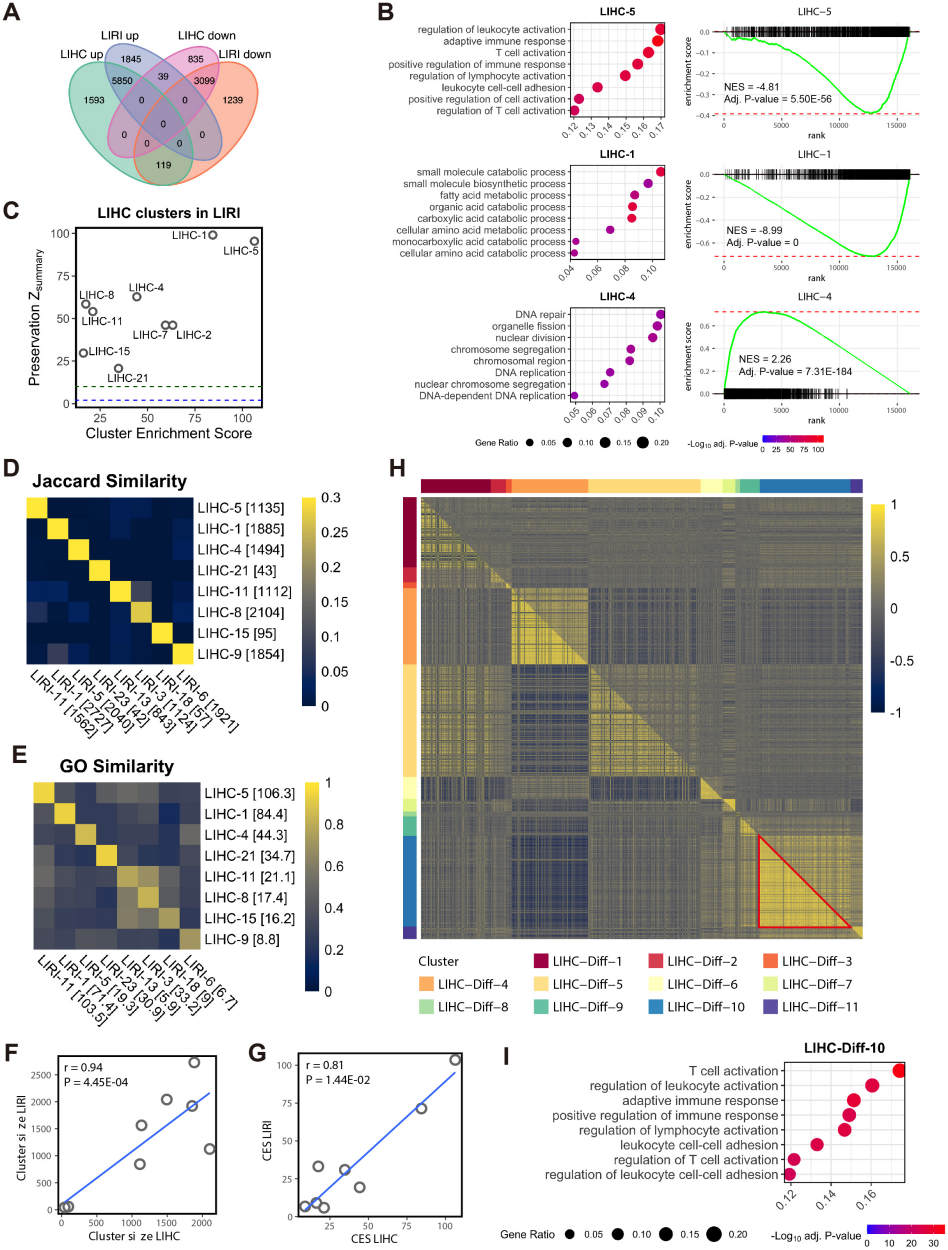
Co-expressed genes associated with immune activities are suppressed in HCC. **(A)** Venn diagram shows the overlap of the DEGs between the LIHC and LIRI cohorts. **(B)** GSOA and GSEA of the WGCNA clusters LIHC-5, LIHC-1, and LIHC-4. For GSEA, genes in the LIHC cohort were sorted based on log_2_ fold change in tumor vs. normal in descending order. **(C)** Evaluation of LIHC co-expression clusters in LIRI cohort. To prioritize biologically significant clusters, only clusters with a CES > 10 are shown (n = 9). Y-axis indicates the module preservation statistics Z_summary_, whereas X-axis indicates the cluster enrichment score. Blue dash line, Z_summary_ = 2; green dash line, Z_summary_ = 10. **(D)** Jaccard similarity between the selected co-expression clusters from LIHC and LIRI cohorts. The number in brackets indicates the size of the cluster. **(E)** GO semantic similarity between the selected co-expression clusters from LIHC and LIRI cohorts. The number in brackets indicates the CES of the clusters. **(F)** Pearson’s correlation between the size of the matched LIHC and LIHC co-expression clusters (n = 8 for each). Each dot represents a pair of matched clusters in **(D, E)**. Data normality was tested by the Shapiro-Wilk test. **(G)** Pearson’s correlation between the CES of the matched LIHC and LIHC co-expression clusters (n = 8 for each). Each dot represents a pair of matched clusters in **(D, E)**. Data normality was tested by the Shapiro-Wilk test. **(H)** Heatmap shows differentially co-expressed genes between normal and tumor samples in the LIHC cohort, as evaluated by Pearson’s correlation coefficient. Upper triangular matrix, correlation in tumor samples; lower triangular matrix, correlation in normal samples; red triangle, correlations between the genes in LIHC-Diff-10 cluster. **(I)** GSOA of the differential co-expression cluster LIHC-Diff-10.

For GSE156337, the processed data (Seurat object) for liver myeloid cells were downloaded from the link given in the original publication (HCC_mye.h5ad, related to **Figure 1** in the original paper, see https://doi.org/10.17632/6wmzcskt6k.1) (39). Four types of tumor-associated macrophages (TAMs), i.e., *FOLR2* ^+^ TAMs (tissue-resident macrophages, TRMs; n = 1,063), *FOLR2* ^+^ TAMs (monocyte-derived macrophages, MDMs; n = 504), *SPP1* ^+^ TAMs (n = 821), and *MT1G* ^+^ TAMs (n = 656) were analyzed. The top 25 markers for the above four macrophage subsets were obtained from the supplemental table deposited in the GEO and were tested for significance of overrepresentation in the top 25 markers for *C1QA* ^+^, *SPP1* ^+^, and *VCAN* ^+^ Mφ identified in the discovery data by hypergeometric testing with Benjamini-Hochberg correction.

For both the discovery and validation cohorts, cells were visualized in the UMAP (Uniform Manifold Approximation and Projection) (41) space based on the first 40 PCs.

### 2.5 Deconvolution cell type in bulk RNA-seq based on scRNA-seq reference

The CIBERSORTx (42) was used to estimate the relative proportion of different immune cell subpopulations in liver immune cells. Using the scRNA-seq discovery cohort as the reference, the expression profiles of liver myeloid cell subsets (*C1QA* ^+^ Mφ, *SPP1* ^+^ Mφ, *VCAN* ^+^ Mφ, cycling Mφ, and DCs) and T cell subsets (*CD8* ^+^ CTLs, *CD4* ^+^ Tregs, memory, tissue-resident, and cycling T cells) were extracted from the full dataset and analyzed separately to estimate the relative content of the corresponding cell type in LIHC and LIRI cohorts, respectively. The separation of myeloid cells and T cells is to ensure the identification of the most discriminative markers for deconvolution. In addition, we also performed CIBERSORTx with the full scRNA-seq discovery cohort with major cell annotation as reference (i.e., without annotating myeloid and T cell subsets) to estimate the fraction of myeloid cells and T cells, respectively. The CIBERSORTx program embedded in the Docker (https://www.docker.com/) was performed in a command line manner, and the batch correction (S-mode, for 10X genomics data) was enabled. The resulting relative cell fractions (**Supplementary Table S6**) were used to analyze the survival risk of the cell content for prognosis (see below section).

### 2.6 Differential expression analysis

For bulk RNA-seq data LIHC and LIRI, differential expression analysis was carried out by the R package DESeq2 (v1.34.0) (17). For both LIHC and LIRI cohorts, after filtering out lowly expressed genes, the rest protein-coding genes were analyzed based on the read count values, comparing their expression between tumors and adjacent normal tissues. The Benjamini-Hochberg procedures were used to correct the p-values. The significance threshold for differentially expressed genes (DEGs) was set as the adjusted p-value < 0.05, with the log_2_ fold change above zero for up-regulation and below zero for down-regulation in tumors (**Supplementary Table S1**). Similarly, the RNA-seq data of HCC-TAMs was analyzed using the same pipeline.

For scRNA-seq data, differential expression analysis was carried out using the Seurat function *FindMarkers()*, with the settings of the adjusted p-value < 0.05 and the average log_2_ fold change > 0 for the identification of the macrophage markers within the liver myeloid subsets and the T cell markers with the liver T cell subsets (**Supplementary Table S5**).

### 2.7 Single-cell regulatory analysis

The single-cell regulatory analysis of liver cell subsets was performed using the pySCENIC pipeline (the Python package pyscenic v0.11.2) (43) based on the scRNA-seq discovery cohort. First, the preprocessed count matrix including 23,605 genes of the total 18,724 cells was further processed by the *geneFiltering()* function in the R SCENIC package (v1.2.4) to filter out lowly expressed genes. Then, the rest 10,641 genes were used to build a GCN by GRNBoost (the Python package arboreto v0.1.6) in Python (v3.7.11). The gene filtering and the use of GRNBoost were to ensure that the program can be finished in due time. Based on the established GCN, the transcription factor binding motif analysis was performed based on the motif collection file hg38:refseq-r80:10kb_up_and_down_tss.mc9nr.feather. The enrichment of the candidate transcription factors (26) was estimated by the AUCell method provided in the pySCENIC pipeline. Finally, a total of 254 TFs were significantly enriched in at least one of the cell types in liver scRNA-seq discovery data (**Supplementary Table S7**).

### 2.8 Trajectory analysis

The trajectory analysis was applied by the R package monocle3 (v1.0.0) (44) to study the trajectory of the differentiation of the cells in the myeloid cell subsets and the T cell subsets, respectively. To determine the starting point of the pseudo time of cell differentiation, the cell that expressed the highest *CD14* was selected as the root for myeloid cells, and the cell that expressed the lowest *CD3E* was selected as the root for T cells.

### 2.9 Cell-cell interaction analysis

CellChat (v1.1.3) (45), an R package for inferring the strength of intercellular communication was applied to the scRNA-seq discovery cohort. Based on the human ligand-receptor databases embedded in the package, the overexpressed genes and the interactions between cell types were identified, followed by the computation of communication probability.

### 2.10 Functional analysis of co-expression gene sets and scRNA-seq cell populations

For bulk RNA-seq data LIHC and LIRI, gene set overrepresentation analysis (GSOA, based on gene ontology) and gene set enrichment analysis (GSEA) were applied to investigate the biological implication of the (differential) co-expression clusters. The analyses were conducted by the R package clusterProfiler (v4.2.1) (46) and fgsea (v1.20.0) (47), respectively. For GSOA, the background was set as the 15,000 genes for co-expression analysis. Here, we defined cluster enrichment score (CES) as the absolute log-transformed (base 10) adjusted p-value of the most significant GO term associated with a co-expression cluster, to help prioritize the most biologically meaningful co-expression clusters (**Supplementary Table S3**). GO terms with a Benjamini-Hochberg adjusted p-value < 0.05 were considered significant (**Supplementary Table S3**). Top-ranked and representative GO terms were selectively shown as dot plots. Similarly, DEGs obtained from the scRNA-seq discovery cohorts (**Supplementary Table S5**) were analyzed by GSOA (**Supplementary Table S8**). For GSEA, the analyzed genes by DESeq2 were sorted by the log_2_ fold change of tumor vs. normal from high to low, followed by the examination of the distribution of the (differentially) co-expressed genes in the ranking list. Here, a positive normalized enrichment score (NES) indicates that the (differential) co-expression cluster is up-regulated (activated) in tumors, and vice versa for negative NES for inhibition (**Supplementary Table S4**). For RNA-seq data of HCC-TAMs, GSEA was analyzed in a similar manner to associate the hallmark gene sets obtained from MSigDB (48) with sh*SPP1* effects.

In order to compare the biological similarity between co-expression clusters from LIHC and LIRI, we calculated the semantic similarity between these clusters based on the significant GO biological processes terms, using the function *mgoSim()* provided in the R package GOSemSim (v2.20.0) (49). In addition, the Jaccard index defined as the intersection relative to the union of two gene sets was used to measure the similarity between co-expression clusters.

For scRNA-seq data, we applied single-sample GSEA (ssGSEA) (50) to infer the overrepresentation of the (differential) co-expression clusters in the scRNA-seq discovery data based on the preprocessed read count matrix. This analysis was carried out by the R package GSVA (v1.42.0) (51). In addition, to understand the functional heterogeneity of liver macrophages and DCs, a list of functional markers for macrophages and DCs was obtained from previous studies (52–54), and their expression was tested in the scRNA-seq discovery cohort by *AddModuleScore()* function.

### 2.11 PROGENy pathway analysis

PROGENy (Pathway RespOnsive GENes for activity inference) (55) was used to analyze the 14 cancer-related pathway activities of each cell type in the scRNA-seq dataset and the *SPP1* inhibition in HCC-TAMs. For scRNA-seq data, the average count for each cell type was calculated, and the cell type expression was normalized and scaled by Seurat. Pathway activity was evaluated using the function *progeny()* in the R package progeny (v1.22.0). For HCC-TAMs data, log2 fold changes of genes in sh*SPP1* HCC-TAMs relative to shControls were used as input, and the pathway activity was calculated using the R package decoupleR (v2.6.0) (56) based on the PROGENy pathway signatures. Similarly, transcription factor activity for sh*SPP1* HCC-TAMs was calculated based on the CollecTRI network – a comprehensive resource containing a curated collection of TFs and their transcriptional targets compiled from 12 different resources (57).

### 2.12 Survival analysis

Survival analysis was performed to study the association of liver myeloid cell subsets and T cell subsets to patients’ survival. For this, cell type abundances (in percentage, see **Supplementary Table S6**) predicted by the CIBERSORTx were used as the input, followed by the univariate Cox regression model and the Kaplan-Meier (KM) survival analysis based on the clinical information of LIHC and LIRI cohorts. For the LIHC cohort, clinical information was retrieved from the TCGA Pan-Cancer Clinical Data Resource (TCGA-CDR) (58). For KM analysis, patients were segregated into high and low groups for each cell type based on the first quartile of the CIBERSORTx abundance. For both survival analyses, cell types with a p-value less than 0.05 were regarded as significantly associated with survival outcomes. The survival analysis was performed by the R package survival (v3.2-13).

### 2.13 Human primary CD8+ T cell culture and Western blot analysis

Human primary *CD8* ^+^ T cells were purchased from Creative Biolabs. *CD8* ^+^ T cells were maintained with RPMI 1640 (R2405, Sigma-Aldrich) supplemented with 10% fetal bovine serum (FBS, F7524, Sigma-Aldrich), 1% P/S (P4333, Sigma-Aldrich), IL-2 (PHC0026, Thermo Fisher), and DynabeadsTM Human T-Activator CD3/CD28 (11161D, Thermo Fisher). 2x10^6^ cells were seeded into 6 well plate and treated purified SPP1 (ab281819, Abcam) at 400ng/ml concentration. The whole cell lysate was prepared with CelLytic M (C2978, Sigma-Aldrich) buffer and prepared with 2x Laemmli Sample Buffer (1610737, Biorad) at 10µg protein lysate. SDS PAGE was performed Mini-PROTEAN^®^ TGX™ Precast Gels (Bio-Rad) and transferred using Trans-Blot^®^ Turbo™ Transfer System (Bio-Rad). CD44 (ab254530, Abcam), and GAPDH (ab8245, Abcam) were blotted as primary antibodies overnight. Secondary antibodies, Goat Anti-Rabbit HRP (ab205718) and goat anti-mouse IgG-HRP (ab97265, Abcam) were blotted for one hour. The protein band was detected with ImageQuantTMLAS 500 (29-0050-63, GE).

### 2.14 Statistical analysis

Data normality was evaluated by the Shapiro-Wilk test. For CIBERSORTx results, statistical differences between tumors and normal tissues were estimated by Wilcoxon rank-sum test. All statistical analyses were done by R (v4.1.2).

## 3 Results

### 3.1 Co-expressed genes associated with immune activities are suppressed in HCC

This study was initiated by exploring two bulk RNA-seq HCC cohorts, LIHC and LIRI. Through differential expression analysis comparing tumor and adjacent normal tissues, we observed a substantial overlap in differentially expressed genes (DEGs) between the LIHC and LIRI cohorts, indicating consistent molecular alterations (**Figure 1A**; **Supplementary Table S1**). Utilizing weighted co-expression network analysis (WGCNA), which identifies co-expressed genes based on gene-gene correlations (21), we identified 22 co-expressed gene clusters in the LIHC cohort, denoted as LIHC-1 to LIHC-22 (**Supplementary Table S2**). Subsequent gene ontology (GO)-based gene set overrepresentation analysis (GSOA) highlighted the remarkable associations of genes in LIHC-5, LIHC-1, and LIHC-4 with immune activities, metabolic processes, and cell proliferation, respectively (**Figure 1B**; **Supplementary Table S3**). Gene set enrichment analysis (GSEA) further illustrated the up-regulation of LIHC-4 and down-regulation of LIHC-5 and LIHC-1 in tumors compared to normal tissues, as observed by the signed normalized enrichment score (NES) (**Figure 1B**; **Supplementary Table S4**). This suggests an overall activation of cell proliferation (possibly malignant cells) and suppression of immune and metabolic processes in HCC tumors.

Analysis of the LIRI cohort independently yielded concordant results with the LIHC cohort. Among the 23 identified co-expressed gene clusters in the LIRI cohort (LIRI-1 to LIRI-23; **Supplementary Table S2**), three clusters (LIRI-11, LIRI-1, and LIRI-5) exhibited similar biological implications and activation/suppression with those in the LIHC-5, LIHC-1, and LIHC-4 clusters, respectively (**Supplementary Figure S1A**, **Supplementary Tables S3**, **S4**). To confirm this correspondence, we assessed the reproducibility of the LIHC clusters in the LIRI cohort using a summarized statistics Z_summary_ for network cluster preservation (23). Nine LIHC clusters exhibited both strong reproducibility (Z_summary_ > 10) in the LIRI data and high biological relevance (cluster enrichment score, CES > 10; see Methods), particularly LIHC-5, LIHC-1, and LIHC-4 (**Figure 1C**). The robust biological relevance of these three pairs of co-expression clusters (**Supplementary Figure S1A**, **Supplementary Table S3**) and their high reproducibility in LIRI (**Figure 1C**) established immune activities, metabolic processes, and cell proliferation as the foremost biological processes in HCC.

Apart from the major three pairs of high consensus co-expression clusters, we also identified five pairs of clusters from each cohort also showing high correspondence based on the Jaccard index for the overlapped gene members (**Figure 1D**) and the GO semantic similarity for biological relevance (**Figure 1E**; **Supplementary Figure S1B**). These pairs included LIHC-21 and LIRI-23 associated with defense response (both suppressed), LIHC-11 and LIRI-13 associated with extracellular matrix (ECM) and collagen (both suppressed), LIHC-8 and LIRI-3 associated with ECM, LIHC-15 and LIRI-18 associated with angiogenesis (both non-directional), and LIHC-9 and LIRI-6 associated with Golgi organization and histone modification (both suppressed). Furthermore, significant correlations were observed between LIHC and LIRI in terms of the size and CES of these eight pairs of clusters (**Figures 1F, G**). In summary, by jointly analyzing transcriptomic data from two independent cohorts including, LIHC and LIRI, these analyses identified and validated consensus co-expression networks that are linked to disease-related biological processes in HCC.

To further highlight the dysregulated biological processes between the HCC tumors and normal tissues, we conducted a differential gene co-expression analysis (24) in the LIHC cohort. This analysis identified eleven differentially co-expressed gene clusters, wherein genes were exclusively co-expressed in either normal tissues or HCC tumors (**Figure 1H**; **Supplementary Table S2**). Notably, one of these clusters, LIHC-Diff-10, which was associated with immune activities (**Figure 1I**; **Supplementary Table S3**), was found to exist only in normal tissues but not in HCC tumors (red triangle in **Figure 1H**). Additionally, the LIHC-Diff-9 cluster, linked to immune responses and type I interferon (**Supplementary Figure S2**, **Supplementary Table S3**), was exclusively co-expressed in normal tissues (**Figure 1H**). These findings suggest the suppression of immune responses in HCC tumors, aligning with our prior observations.

### 3.2 The consensus immunosuppression co-expression cluster is dominated by immunoregulatory transcription factors

**Figure 2:**
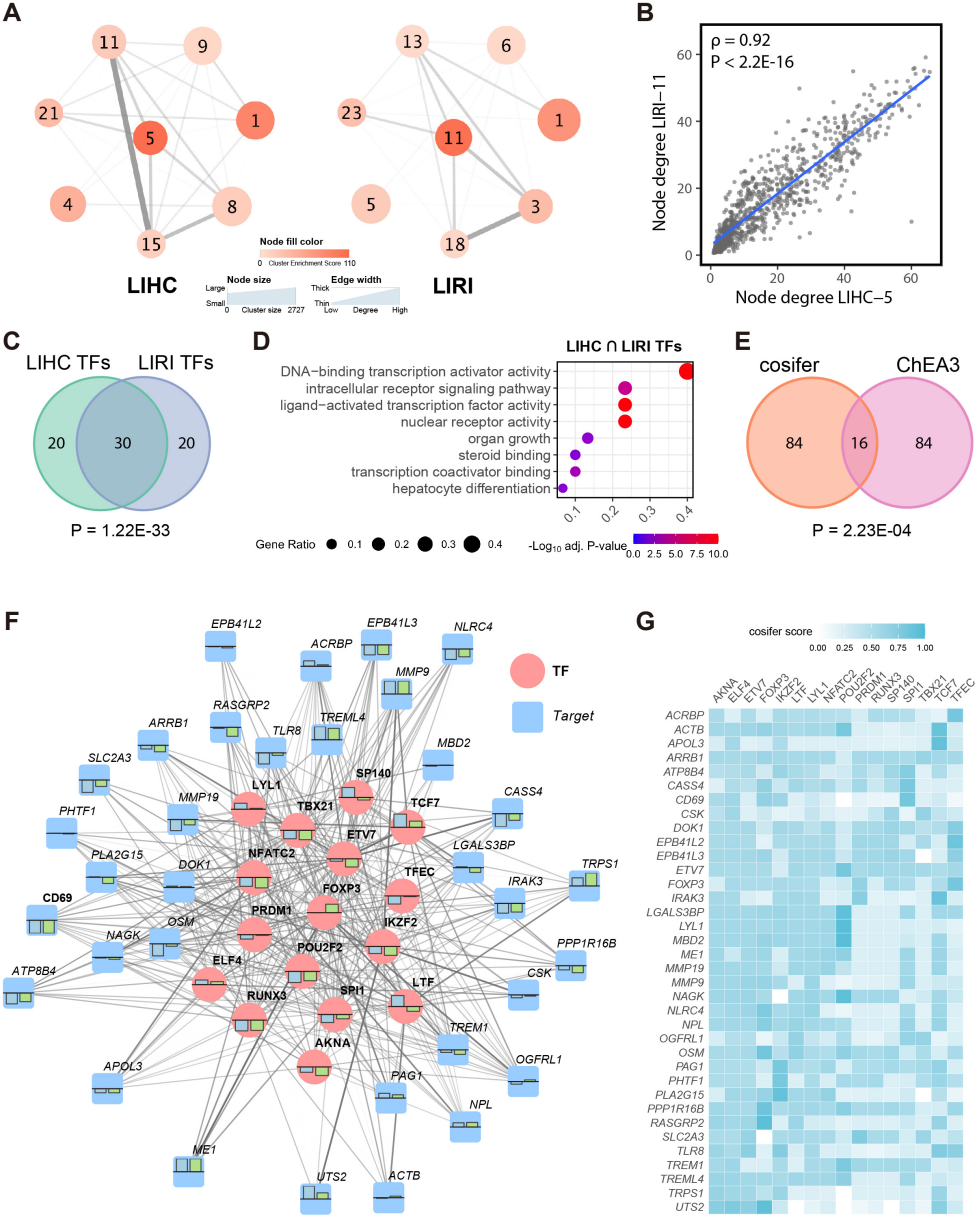
The consensus immunosuppression co-expression cluster is dominated by immunoregulatory transcription factors. **(A)** WGCNA network structure of the selected corresponding co-expression clusters (see **Figures 1D, E**) from LIHC and LIRI cohorts, respectively, presented at the cluster level. Edges were evaluated by the average connections between all genes in different clusters based on the adjacency matrix. **(B)** Spearman’s correlation between the node degree of the common genes (n = 921) in LIHC-5 and LIRI-11 clusters. **(C)** Venn diagram shows the overlap between the top 50 TFs in LIHC and LIRI GRNs. **(D)** GSOA of the intersected TFs in **(C)**. **(E)** Venn diagram shows the overlap between the top 100 TFs in the cosifer-combined GRNs and the results of ChEA3. **(F)** The sub-regulatory network for the consensus immunosuppression cluster. Blue and green bars show the log_2_ fold change of the TFs/targets in the LIHC and LIRI cohort, respectively, with the upper bound as 1 and lower bound as -1. For simplicity, only the 16 TFs in **(E)** and their 3 most connected targets are shown. **(G)** Heatmap shows the edge weights presented in **(F)**, defined by the cosifer scores.

To obtain a comprehensive overview of the consensus portion of the two co-expression networks, matched co-expression clusters (n = 8 from each) were visualized separately per cohort (**Figure 2A**), with clusters aligned according to their corresponding counterparts (see **Figures 1D, E**). Notably, these eight pairs of co-expression clusters encompassed more than half of the total 15,000 analyzed genes (in LIHC, 9,722 genes in the eight clusters, 65%; in LIRI, 10,316 genes in the eight clusters, 69%), providing a representation of the global transcriptional landscape in HCC. Clear links were observed between the immunosuppression clusters LIHC-5/LIRI-11 and other biologically significant clusters, underscoring the pivotal role of these immunosuppression clusters in both networks. Furthermore, LIHC-5 and LIRI-11 exhibited similar topological characteristics, evidenced by a strong correlation between the degree (the sum of weighted connections) of the common genes (n = 921) in LIHC-5 and LIRI-11 (**Figure 2B**). Collectively, these findings suggest that the immunosuppression co-expression clusters LIHC-5 and LIRI-11 were highly consistent and represented the most central and biologically relevant clusters in the TCGA-LIHC and ICGC-LIRI-JP co-expression networks, respectively.

The remarkable correspondence between LIHC-5 and LIRI-11 prompted the establishment of a consensus gene regulatory network (GRN) for the immune system in HCC. To achieve this, the cosifer algorithm (25) was applied to both the LIHC and LIRI transcriptomics data, independently forming two global GRNs. The cosifer is an ensemble gene regulatory network inference algorithm that integrates multiple state-of-the-art gene regulatory network inference methods, generating a consensus GRN by harnessing the wisdom of crowds (59). For each cohort, eight meta-GRNs were constructed using different GRN inference algorithms and subsequently combined (see Methods) to create a cohort-specific GRN. Examination of the top 50 transcription factors (TFs) in each cohort-specific GRN (evaluated by the network degree, i.e., the sum of the regulatory strength from a TF to its targets) revealed a significant overlap of TFs (n = 30, p = 1.22E-33, hypergeometric testing) (**Figure 2C**), indicating substantial similarity among the key TFs in the LIHC and LIRI GRNs. Furthermore, these 30 TFs were significantly associated with regulatory and hepatocyte-related processes (**Figure 2D**), further supporting their relevance in HCC. Overall, the establishment of cohort-specific GRNs revealed a high degree of correspondence between LIHC and LIRI at the gene regulatory level.

Given this strong concordance, we integrated the meta-GRNs from LIHC and LIRI using cosifer to create a unified global GRN, capturing consensus information from both cohorts. Given the established importance of immune clusters in both cohorts, we focused on constructing a sub-GRN for the consensus immunosuppression cluster, defined as the intersection of genes between LIHC-5 and LIRI-11 (LIHC-5 ∩ LIRI-11, n = 921), from the global consensus GRN. Based on network degree, the top 100 highest-ranked TFs for this immunosuppression sub-GRN were prioritized. These data-driven, computation-based TFs were further refined by intersecting with the top 100 TFs for the consensus immunosuppression cluster predicted by ChEA3, a knowledge-based TF prediction algorithm (33), resulting in 16 highly credible TFs (p = 2.23E-04, hypergeometric testing) for the consensus immune cluster (**Figure 2E**).

Among the 16 TFs, several have previously been linked to HCC and immune activities, including PRDM1 in *CD8* ^+^ T cells (60), SPI1 in myeloid cell development, FOXP3 in regulatory T cells (61), and TCF7 in HCC (62). The 16 TFs, along with their top three connected targets, were visualized as a network to portray the entire sub-GRN for the consensus immunosuppression cluster (**Figures 2F, G**). Generally, the TFs and targets consistently exhibited differential expression in tumors compared to normal tissues in both LIHC and LIRI cohorts. Furthermore, a higher number of genes and targets were down-regulated in this sub-GRN, further corroborating the suppression of the immune response in HCC.

### 3.3 Single-cell RNA-sequencing analysis associated the immune co-expression network with liver macrophages

**Figure 3:**
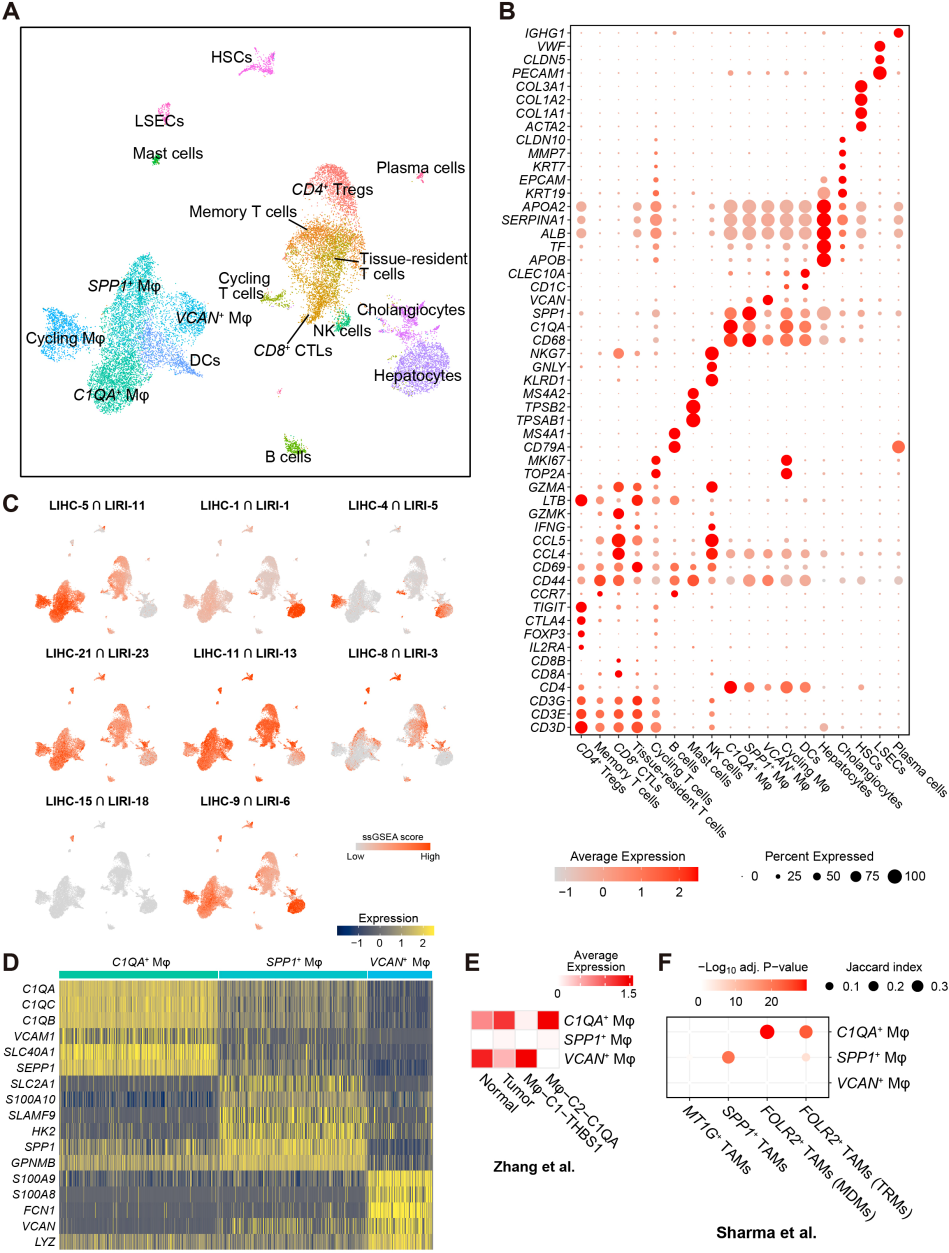
Single-cell RNA-sequencing analysis associated the immune co-expression network with liver macrophages. **(A)** Two-dimensional UMAP visualization of the liver cancer cells in the scRNA-seq GSE166635 cohort. **(B)** Expression of markers for cell types presented in **(A)**. **(C)** Enrichment of the intersected genes between LIHC and LIRI consensus co-expression clusters (see **Figures 1D, E**) in the scRNA-seq discovery cohort evaluated by ssGSEA. **(D)** Heatmap shows the expression of the top-ranked markers for *C1QA* ^+^, *SPP1* ^+^, and *VCAN* ^+^ liver macrophages. **(E)** Expression of the markers for *C1QA* ^+^, *SPP1* ^+^, and *VCAN* ^+^ macrophages in the macrophage subsets Mφ-C1-THBS1 and Mφ-C2-C1QA in GSE140228 cohort (8). **(F)** Overrepresentation of the markers for *C1QA* ^+^, *SPP1* ^+^, and *VCAN* ^+^ macrophages in the macrophage subsets *FOLR2* ^+^ TAMs (TRMs, tissue-resident macrophages), *FOLR2* ^+^ TAMs (MDMs, monocyte-derived macrophages), *SPP1* ^+^ TAMs, and *MT1G* ^+^ TAMs in GSE156337 cohort (39). Significance was evaluated by hypergeometric testing followed by Benjamini-Hochberg correction (adjusted p-value).

As the bulk RNA-seq cohort cannot precisely capture gene expression and co-expression in specific cell types, we utilized a publicly available scRNA-seq cohort, GSE166635, as the discovery cohort for HCC (13). After performing the quality control and preprocessing, which included the removal of lowly expressed genes, unqualified cells, and droplets (**Supplementary Figures S3A-E**) (see Methods), a total of 18,724 cells were clustered into 18 distinct cell types based on the expression of 23,605 genes (**Figure 3A**). Cell clusters were annotated based on previous scRNA-seq studies in HCC (6–8, 10, 38) and the Tabula Sapiens (63). These included B cells (*MS4A1*, *CD79A*), dendritic cells (DCs; *CLEC10A*, *CD1C*), hepatic stellate cells (HSCs; *ACTA2*, *COL1A1*, *COL1A2*, *COL3A1*), liver sinusoidal endothelial cells (LSECs; *PECAM1*, *CLDN5*, *VWF*), mast cells (*MS4A2*, *TPSB2*, *TPSAB1*), macrophages (Mφ; *CD68*), T cells (*CD3D*, *CD3E*, *CD3G*), plasma cells (*IGHG1*, *CD79A*), hepatocytes (including malignant cells; *ALB*, *TF*, *APOB*), cholangiocytes (*KRT19*, *EPCAM*), and natural killer cells (NK cells; *NKG7*, *GNLY*, *KLRD1*) (**Figure 3B**).

Furthermore, macrophage subsets were annotated based on highly expressed markers, including *C1QA* ^+^ Mφ, *SPP1* ^+^ Mφ, *VCAN* ^+^ Mφ, and cycling Mφ (*TOP2A*, *MKI67*) (**Figure 3B**). DCs formed a major myeloid cluster (**Figure 3A**). Similarly, five distinct T cell subsets were identified in the large T cell cluster. Based on the markers in **Figure 3B** (9), these T cell subsets were annotated as *CD4* ^+^ Tregs (*CD4*, *TIGIT*, *CTLA4*, *FOXP3*, *IL2RA*), *CD8* ^+^ cytotoxic T (lymphocyte) cells (*CD8* ^+^ CTLs; *CD8A*, *CD8B*, *GZMK*, *CCL4*, *CCL5*), tissue-resident T cells (*CD69*), memory T cells (*CD44*), and cycling T cells (*TOP2A*, *MKI67*).

For the eight pairs of co-expression clusters obtained from the bulk RNA-seq analysis (see **Figures 1D, E**), we associated the overlapping genes between the corresponding clusters with the cells in the scRNA-seq data using single-sample gene set enrichment analysis (ssGSEA) (50). While previous results from gene set overrepresentation analysis (GSOA) associated LIHC-5 and LIRI-11 primarily with T cell activities and adaptive immune responses (**Figure 1B**; **Supplementary Figure S1A**), we found that genes in the consensus immunosuppression cluster (LIHC-5 ∩ LIRI-11) were predominantly enriched in liver macrophages, with a lesser extent of enrichment in T cells (**Figure 3C**). This observation shifted our focus toward liver macrophages. Additionally, several consensus clusters were enriched in specific cell types (**Figure 3C**; **Supplementary Figure S4A**), including the consensus metabolic cluster (LIHC-1 ∩ LIRI-1) in hepatocytes (including malignant cells), the consensus proliferation cluster (LIHC-4 ∩ LIRI-5) in cycling cells and a portion of hepatocytes (which could be malignant cells), and the consensus angiogenesis cluster (LIHC-15 ∩ LIRI-18, see **Supplementary Figure S1B**) in LSECs. Moreover, cluster LIHC-Diff-10, which was exclusively co-expressed in normal tissues (**Figure 1H**) and associated with immune activities (**Figure 1I**), was also enriched in macrophages, with a lesser extent of enrichment in T cells (**Supplementary Figures S4B, C**). Another noteworthy observation is the enrichment of LIHC-Diff-11 in HSCs (**Supplementary Figures S4B, C**) – the cells primarily responsible for the accumulation of the extracellular matrix (ECM) in liver fibrosis (64). These genes in the LIHC-Diff-11 cluster were exclusively co-expressed in HCC tumors (**Figure 1H**) and were associated with ECM (**Supplementary Figure S2**), implying a potential role for HSCs regulated by this differential co-expression network in the context of HCC.

The myeloid cell cluster (located at the bottom left in **Figure 3A**) showing the highest relevance to the immunosuppression co-expression cluster (**Figure 3C**), was further subdivided into DCs and four subtypes of liver macrophages. Beyond the proliferating macrophages, three distinct macrophage subsets were annotated: *C1QA* ^+^ Mφ, characterized by high expression of *C1QA*, *C1QB*, *C1QC*, *VCAM1*, *SEPP1*; *SPP1* ^+^ Mφ, characterized by high expression of *SPP1*, *SLC2A1*, *S100A10*; and *VCAN* ^+^ Mφ, characterized by high expression of *FCAN*, *FCN1*, *S100A8*, *S100A9* (**Figure 3D**; **Supplementary Table S5**). The existence of these three Mφ subsets was further validated in two independent scRNA-seq datasets for HCC: GSE140228 (8) and GSE156337 (39). *C1QA* ^+^ Mφ markers were highly expressed in Mφ-C2-C1QA cell cluster and in tumors, while *VCAN* ^+^ Mφ markers were highly expressed in Mφ-C1-THBS1 cell cluster and in normal tissues (**Figure 3E**; **Supplementary Figure S5A**), confirming the presence of *C1QA* ^+^ and *VCAN* ^+^ Mφ subsets in GSE140228 and suggesting distinct functions between these two Mφ subsets. Although *SPP1* ^+^ Mφ markers were not highly expressed in macrophages in GSE140228 (**Figure 3E**, **Supplementary Figure S5A**), they were overrepresented in *SPP1* ^+^ tumor-associated macrophages (TAMs) in GSE156337. While *VCAN* ^+^ macrophage markers were not overrepresented in the GSE156337 cohort (**Figure 3F**; **Supplementary Figure S5B**), *C1QA* ^+^ macrophage markers were significantly overrepresented in *FOLR2* ^+^ TAMs (**Figure 3F**), which were reported as immunosuppressive macrophage subset (39). Overall, our analysis successfully validated the existence of all three identified macrophage populations in two independent cohorts. Furthermore, the absence of *SPP1* ^+^ macrophages in GSE140228 and *VCAN* ^+^ macrophages in GSE156337 suggested a diverse landscape of macrophage subsets within the discovery cohort.

### 3.4 A high fraction of *SPP1* ^+^ macrophages is harmful to HCC patients’ survival

**Figure 4:**
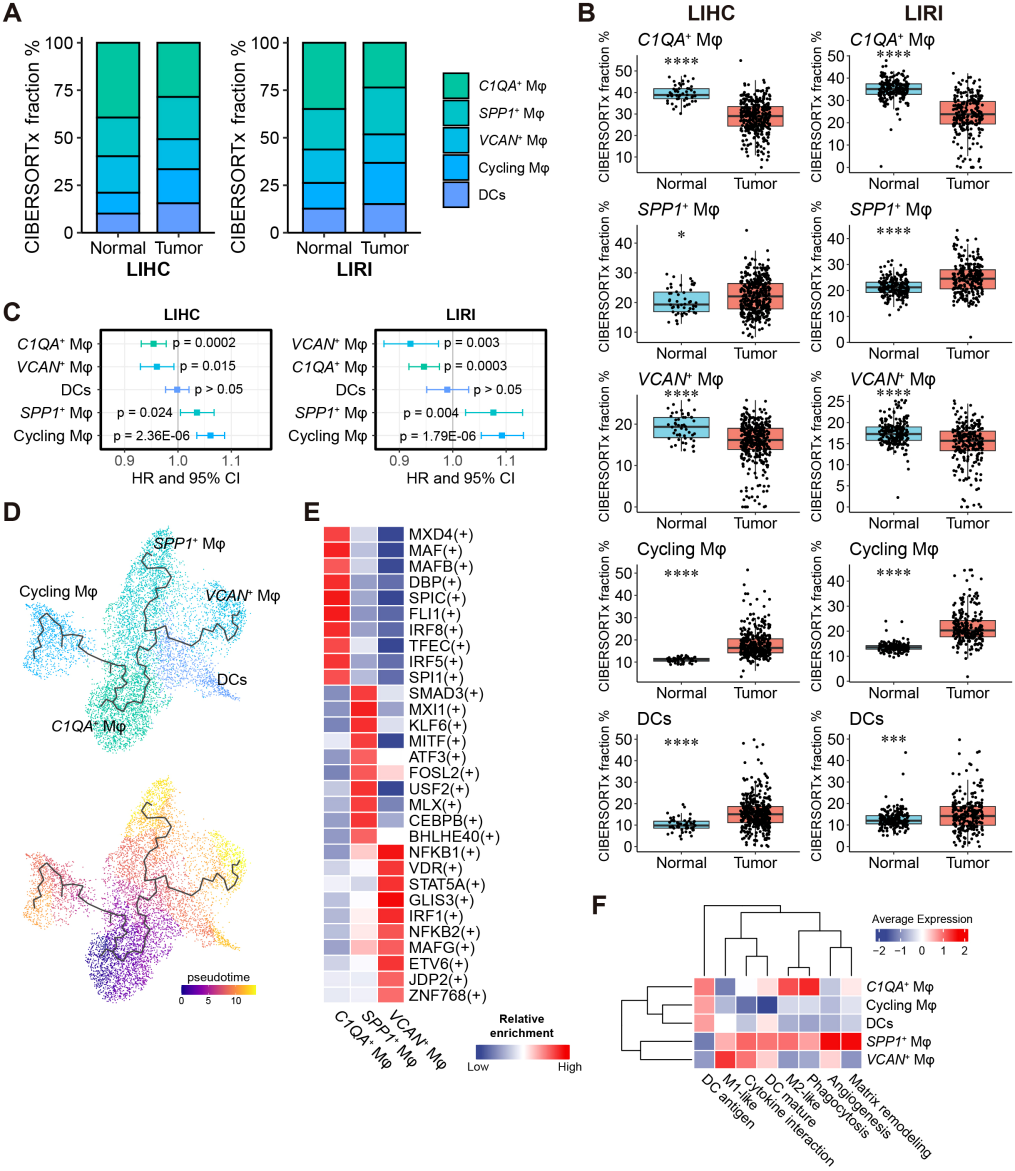
A high fraction of *SPP1* ^+^ macrophages is harmful to HCC patients’ survival. **(A)** Relative fractions of *C1QA* ^+^, *SPP1* ^+^, *VCAN* ^+^, and cycling macrophages and DCs in the LIHC and LIRI cohorts estimated by CIBERSORTx. **(B)** Comparison of the relative fractions of the five subtypes in **(A)** between normal tissues and tumors in LIHC and LIRI cohorts. *p < 0.05, **p < 0.01, ***p < 0.001, ****p < 0.0001 by Wilcoxon rank-sum test. **(C)** Forest plot shows the hazard ratio (HR) and 95% confidence interval (CI) of the association of relative macrophage/DC content to patients’ survival, evaluated by univariate Cox survival model. **(D)** Cell differentiation trajectories between macrophage subtypes and DCs. The zero-pseudo time was determined as the cell expressed the highest monocyte marker CD14. **(E)** The top 10 enriched TFs for *C1QA* ^+^, *SPP1* ^+^, and *VCAN* ^+^ macrophages identified by SCENIC. **(F)** Average (relative) expression of predefined functional markers in the macrophages/DCs from the GSE166635 cohort.

The analysis of the relative proportion of immune cells in bulk RNA-seq data using the CIBERSORTx method (42), with scRNA-seq data as the reference, revealed consistent results between the LIHC and LIRI cohorts (**Figures 4A, B**; **Supplementary Figure S6**, **Supplementary Table S6**). First, we found an increased level of myeloid cells and a decreased level of T cells in tumors over normal samples across the two cohorts (**Supplementary Figure S6**), suggesting an overall immunosuppression of T cells in tumors, which could be regulated by the infiltrated myeloid cells. Among the myeloid cell cluster components (*C1QA* ^+^ Mφ, *SPP1* ^+^ Mφ, *VCAN* ^+^ Mφ, cycling Mφ, and DCs), *C1QA* ^+^ Mφ and *SPP1* ^+^ Mφ were the two most abundant macrophage subsets constituting more than 50% of the total liver macrophages and DCs (**Figure 4A**). Furthermore, *SPP1* ^+^ Mφ, cycling Mφ, and DCs exhibited significantly higher content in tumors, while *C1QA* ^+^ and *VCAN* ^+^ Mφ were reduced in tumor samples in both the LIHC and LIRI HCC cohorts (**Figure 4B**).

To assess the impact of the relative content of liver macrophages and DCs on patient survival, we performed univariable Cox regression analysis. High proportions of *C1QA* ^+^ and *VCAN* ^+^ macrophages were associated with a favorable prognosis (Hazard Ratio HR < 1, p < 0.05), whereas high proportions of *SPP1* ^+^ and cycling macrophages were linked to an unfavorable prognosis (HR > 1, p < 0.05), consistently observed in both the LIHC and LIRI cohorts (**Figure 4C**). The Kaplan-Meier survival analysis further confirmed these findings (**Supplementary Figure S7A**).

Despite the close relationship between DCs and macrophages, trajectory analysis (44) of the myeloid cell cluster revealed differentiation trajectories exclusively between macrophage cell types and not to DCs (**Figure 4D**). The *C1QA* ^+^ macrophages were identified as progenitor cells giving rise to *SPP1* ^+^, *VCAN* ^+^, and cycling macrophages through three distinct trajectories (**Figure 4D**).

To identify cell-type-specific transcription factors, we applied single-cell regulatory network inference and clustering (SCENIC) (43) to the scRNA-seq discovery cohort. A total of 254 TFs were enriched in at least one of the 18 liver cell types (**Supplementary Table S7**). UMAP visualization based on the SCENIC TF-target activity matrix showed six major cell clusters among the 18,724 cells, with most liver cell types clearly separated (**Supplementary Figure S8A**). Nine of the 16 total TFs identified for the consensus immunosuppression cluster (**Figure 2F**) were also significant in the single-cell regulatory analysis and were predominantly enriched in macrophages and T cells (**Supplementary Figure S8B**), reaffirming their relevance. For each of the three major macrophage subsets (*SPP1* ^+^ Mφ, *VCAN* ^+^ Mφ, and *C1QA* ^+^ Mφ), we identified the top 10 most specific TFs as representatives (**Figure 4E**). Notably, *VCAN* ^+^ Mφ appeared to be regulated by pro-inflammatory TFs such as NFKB1, NFKB2, and STAT5A (65), indicating a pro-inflammatory and M1-like phenotype of *VCAN* ^+^ Mφ.

The functional heterogeneity of myeloid cells was explored in detail by analyzing the overall expression of macrophage functional markers obtained from previous studies (52–54) as well as conducting GSOA of the markers for each cell type (**Supplementary Tables S5**, **S8**). *SPP1* ^+^ Mφ displayed an M2-like phenotype, involved in matrix remodeling, angiogenesis, and wound healing (**Figure 4F**, **Supplementary Figure S9A**, **Supplementary Table S8**). Conversely, *VCAN* ^+^ Mφ exhibited an M1-like phenotype, characterized by pro-inflammatory cytokine production (**Figure 4F**; **Supplementary Figure S9A**, **Supplementary Table S8**). Additionally, *C1QA* ^+^ Mφ showed relevance to both an M2-like phenotype and phagocytosis, but not angiogenesis and matrix remodeling, distinguishing from the other M2-like (*SPP1* ^+^) macrophage subset (**Figure 4F**; **Supplementary Figure S9A**; **Supplementary Table S8**). The cycling macrophages, despite being significantly associated with prognosis (**Figure 4C**; **Supplementary Figure S7A**), were less emphasized in our analyses, mainly due to their nondirective developmental trajectory to any other macrophage subtypes (**Figure 4D**) with certain biological functions (**Figure 4F**; **Supplementary Figure S9A**), and absence as a major source of liver macrophages in other HCC studies (6–8, 10, 39).

**Figure 5:**
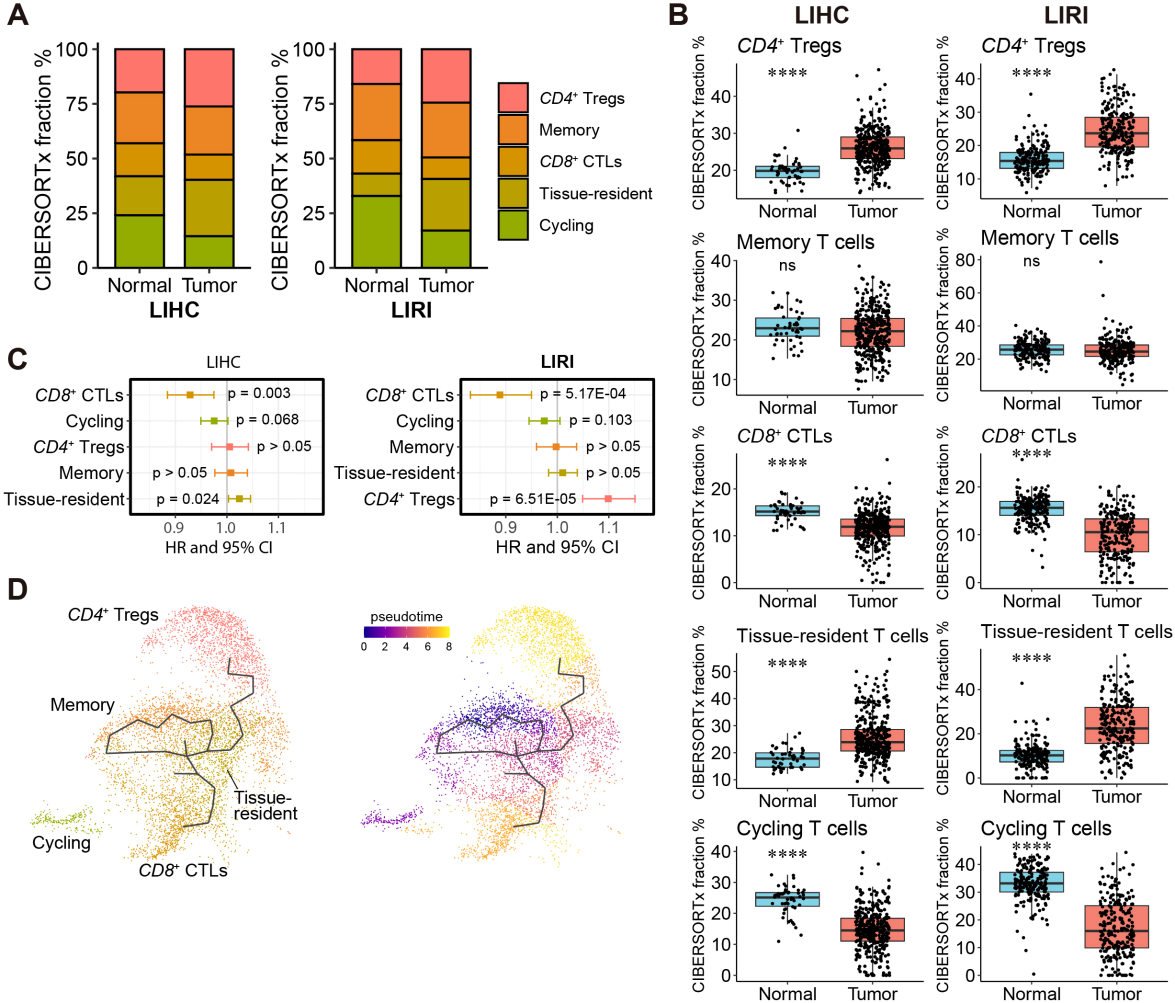
A high fraction of *CD8* ^+^ CTLs is favorable for patients’ survival. **(A)** Relative fractions of *CD4* ^+^ Tregs, *CD8* ^+^ CTLs, memory, tissue-resident, and cycling T cells in the LIHC and LIRI cohorts estimated by CIBERSORTx. **(B)** Comparison of the relative fractions of the five T cell subtypes in **(A)** between normal tissues and tumors in LIHC and LIRI cohorts. *p < 0.05, **p < 0.01, ***p < 0.001, ****p < 0.0001 by Wilcoxon rank-sum test. **(C)** Forest plot shows the hazard ratio (HR) and 95% confidence interval (CI) of the association of relative T cell content to patients’ survival, evaluated by univariate Cox survival model. **(D)** Cell differentiation trajectories between T cell subtypes. The zero-pseudo time was determined as the cell expressed the lowest T cell marker CD3E.

In parallel, we applied the same analytical pipeline to investigate the heterogeneity and prognosis of liver T cells. Consistent results were observed between LIHC and LIRI, showing significantly higher content of *CD4* ^+^ Tregs and tissue-resident T cells and lower content of cycling and *CD8* ^+^ CTLs in liver tumors (**Figures 5A, B**). Among the five T cell subsets, both Cox survival analysis and Kaplan-Meier survival analysis consistently demonstrated a favorable prognosis associated with *CD8* ^+^ CTLs, highlighting their importance for patient survival (**Figure 5C**; **Supplementary Figure S7B**). However, the prognostic significance of the other T cell subsets was inconclusive. GSOA of liver T cell subsets revealed functional heterogeneity of T cell subsets, with *CD8* ^+^ CTLs involved in cytotoxic and cell-killing functions, and cycling T cells related to proliferation (**Supplementary Figure S9B**, **Supplementary Table S8**). Trajectory analysis identified differentiation trajectories of *CD4* ^+^ Tregs and *CD8* ^+^ CTLs originating from memory and tissue-resident T cells, suggesting the latter two as the sources of functional T cells in HCC (**Figure 5D**).

### 3.5 *SPP1* ^+^ macrophages interact with T cells through the SPP1 – CD44 ligand-receptor pair

**Figure 6:**
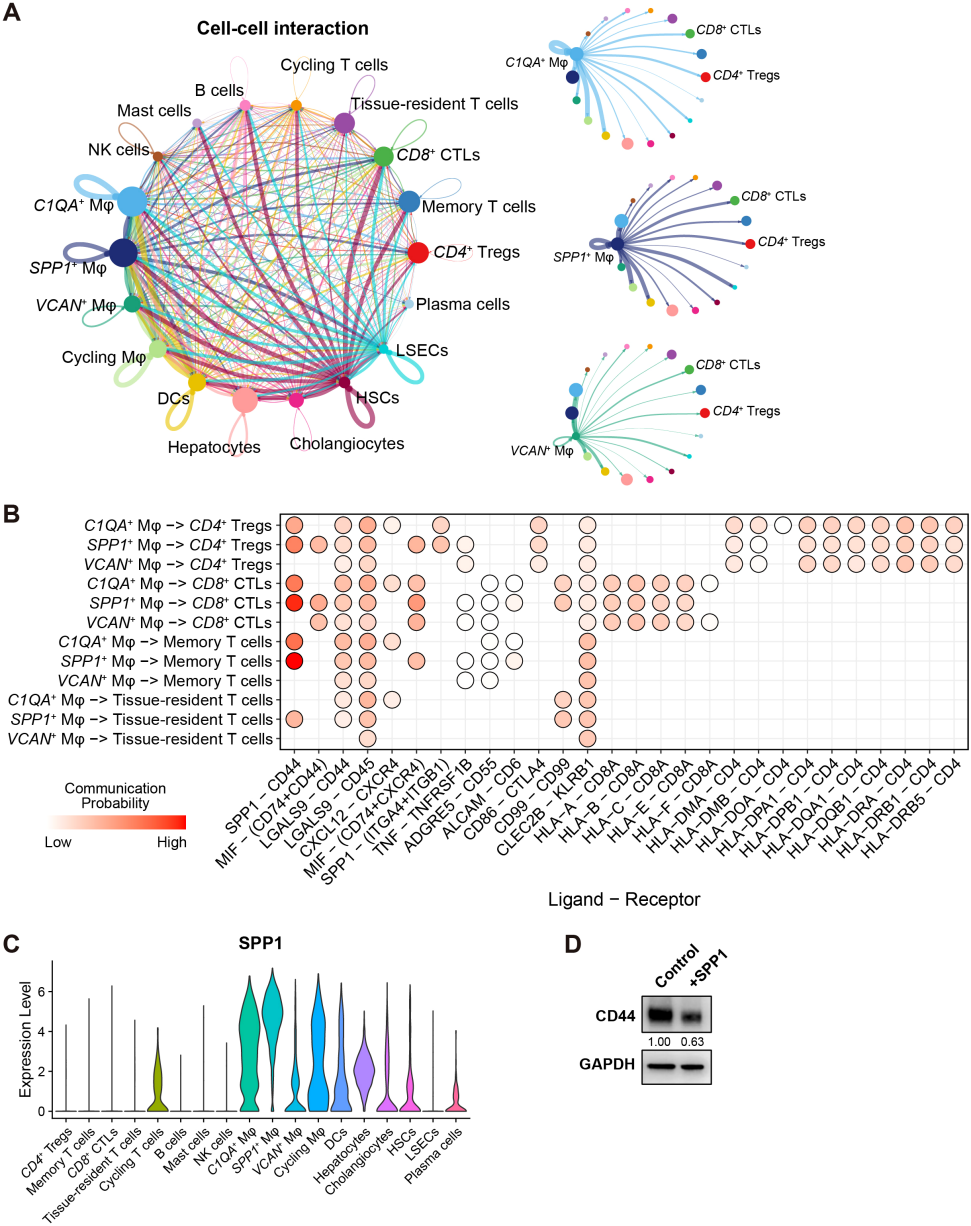
*SPP1* ^+^ macrophages interact with T cells through the SPP1 – CD44 ligand-receptor pair. **(A)** Cell-cell interactions between 18 cell types in HCC. Edges represent the total interaction strength between two cell types; node size indicates the number of cells in one cell type. Interaction maps from *C1QA* ^+^, *SPP1* ^+^, and *VCAN* ^+^ macrophages to the other cell types are shown independently. **(B)** Dot plot shows the communication probability based on the ligand-receptor pair between *C1QA* ^+^, *SPP1* ^+^, *VCAN* ^+^ macrophages, and T cells. Only significant ligand-receptor pairs are shown as dots. **(C)** Violin plots show the expression of *SPP1* in the 18 cell types. **(D)** Western blot shows the level of CD44 in CD8+ T cells treated with 400ng/ml of purified SPP1. Band intensity was normalized with GAPDH.

In the analysis of cell-cell interactions within the scRNA-seq discovery cohort by CellChat (45), it was found that HSCs exhibited strong interactions with other liver cells (**Figure 6A**), consistent with a previous report (38). More importantly, *SPP1* ^+^ Mφ showed significant interactions with LSECs, suggesting a potential anti-inflammatory role. Notably, among the three major liver macrophage subsets, *CD8* ^+^ CTLs and *CD4* ^+^ Tregs were highly influenced by these macrophage subsets through different ligand-receptor pairs, with SPP1 – CD44 ligand-receptor pair as the strongest one (**Figures 6A, B**).

As depicted in **Figure 6C**, *SPP1* was highly expressed in *SPP1* ^+^ Mφ, followed by *C1QA* ^+^ Mφ, but not in *VCAN* ^+^ Mφ. Indeed, the average expression of *SPP1* in *SPP1* ^+^ Mφ was almost 3 folds higher than in *C1QA* ^+^ Mφ (average log_2_ fold change = 1.53, adjusted p-value = 2.27E-236), suggesting that *SPP1* ^+^ Mφ are key contributors to the SPP1 – CD44 ligand-receptor axis, facilitating interactions between macrophages and T cells in HCC. Moreover, a supplement of purified SPP1 protein to primary human *CD8* ^+^ T cells reduced the expression of CD44 – a prominent marker for T cell activation, showing that macrophage-secreted SPP1 regulates *CD8* ^+^ T cell activity in a reversed direction (**Figure 6D**).

In summary, the SPP1 – CD44 ligand-receptor pair plays a critical role in mediating interactions between liver macrophages – particularly *SPP1* ^+^ Mφ, and various T cell subsets in the liver, programming immune responses in the TIME.

### 3.6 Inhibition of *SPP1* in HCC-TAMs drives macrophage transition toward a favorable phenotype

**Figure 7:**
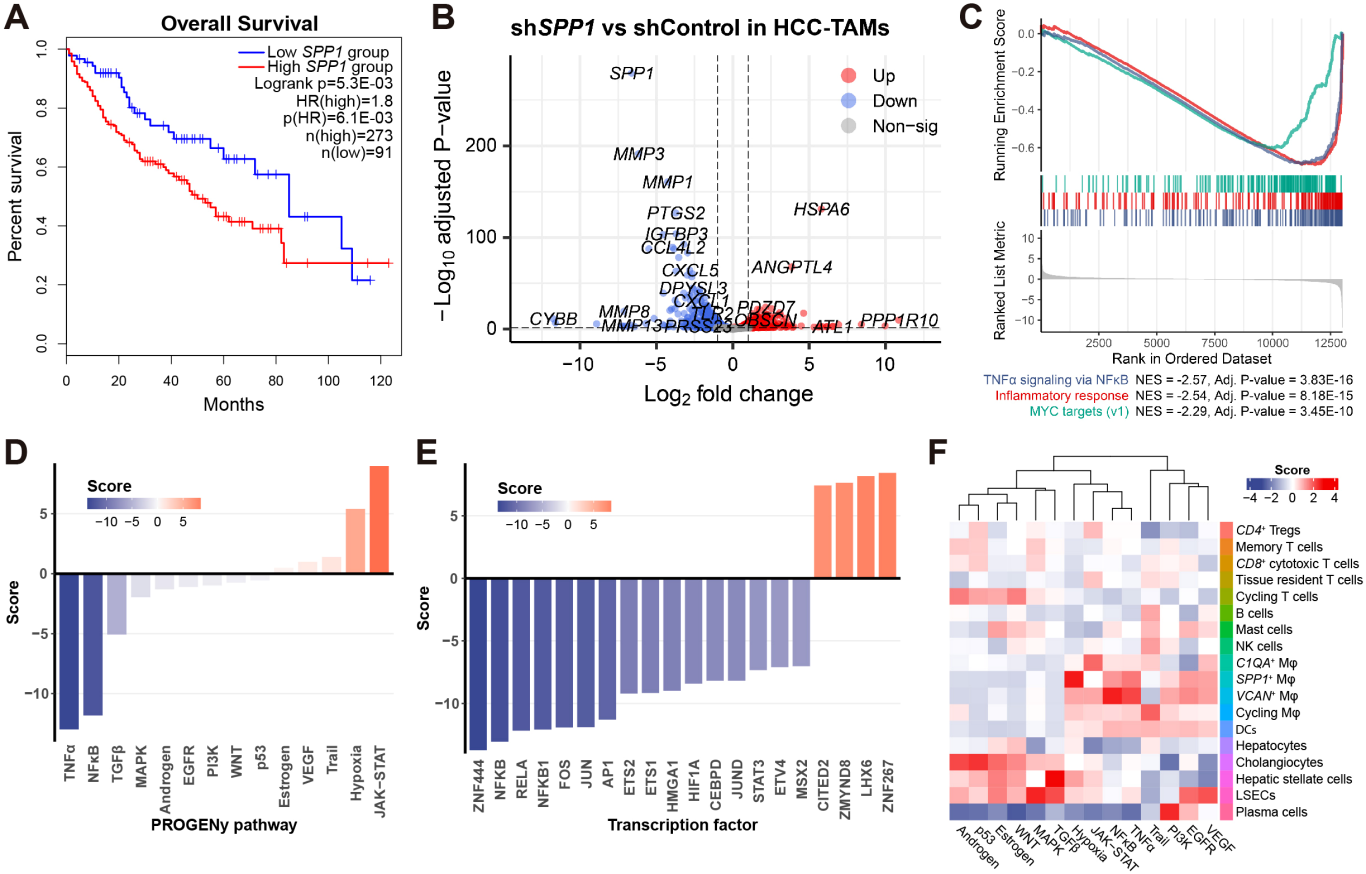
Inhibition of *SPP1* in HCC-TAMs drives macrophage transition toward a favorable phenotype. **(A)** Kaplan-Meier survival analysis (Logrank) and univariate Cox survival analysis (HR) associate the expression of *SPP1* to patients’ survival based on the LIHC cohort. **(B)** Volcano plot shows the dysregulated genes between sh*SPP1* and shControl in HCC-TAMs. **(C)** GSEA plot shows the top-3 most enriched hallmark gene sets associated with sh*SPP1* effects. **(D)** Barplot shows the PROGENy pathway activity of sh*SPP1* relative to control. **(E)** Barplot shows the transcription factor activity of sh*SPP1* relative to control. **(F)** Heatmap shows the relative PROGENy pathway activity of 18 cell types in the HCC scRNA-seq dataset.

Given that *SPP1* is primarily expressed in liver macrophages and hepatocytes (**Figure 6C**), we explored whether the overall expression of *SPP1* in HCC correlates with patient survival. Employing GEPIA2 (66), we conducted Kaplan-Meier survival analysis and univariate Cox survival analysis, both of which provided compelling evidence that elevated levels of *SPP1* are associated with poor patient outcomes (**Figure 7A**), confirming the unfavorable role of *SPP1* expression in HCC.

To further elucidate the molecular mechanisms of *SPP1* in TAMs, we analyzed publicly available data (GSE230666), which involved the inhibition of *SPP1* in HCC-TAMs (sh*SPP1*) along with negative control (shControl) (18). Upon inhibition of *SPP1* in HCC-TAMs, we observed a significant down-regulation of numerous matrix metalloproteinase (MMP) genes (**Figure 7B**), suggesting that *SPP1* plays a role in ECM functions in TAMs, in accordance with the findings in **Figure 4F**. Additionally, GSEA indicated that *SPP1* inhibition led to the attenuation of pro-inflammatory effects in HCC-TAMs (**Figure 7C**). This observation was further substantiated by a parallel analysis based on PROGENy pathway activity (55), which demonstrated the suppression of TNFα and NFκB pathway activities in sh*SPP1* (**Figure 7D**). Similarly, transcription factor analysis revealed a decrease in NFκB activity upon *SPP1* inhibition (**Figure 7E**).

For comparative purposes, we conducted PROGENy pathway analysis on the scRNA-seq dataset of the discovery cohort. This analysis unveiled several noteworthy findings, including the heightened activity of EGFR and VEGF signaling pathways in LSECs, as well as elevated TGFβ signaling in HSCs (the primary cell type associated with liver fibrosis) (**Figure 7F**). Focusing on liver macrophage cell types, we observed a heightened JAK-STAT signaling activity in *C1QA* ^+^ Mφ, hypoxia signaling in *SPP1* ^+^ Mφ, and activation of TNFα and NFκB signaling pathways in *VCAN* ^+^ Mφ (**Figure 7F**). These findings align with our prior results, which demonstrated the association of *SPP1* ^+^ Mφ with responses to hypoxia (**Supplementary Figure S9A**), and *VCAN* ^+^ Mφ with pro-inflammatory responses (**Figures 3E, F**).

Collectively, the inhibition of *SPP1* in HCC-TAMs resulted in the down-regulation of pro-inflammatory signaling pathways (TNFα and NFκB) and relevant transcription factors (NFκB family) (**Figures 7D, E**). This response contrasted with the behavior of *VCAN* ^+^ Mφ. Additionally, *SPP1* inhibition induced an up-regulation of the JAK-STAT signaling pathway (**Figure 7D**), mirroring the pathway activity profile observed in *C1QA* ^+^ Mφ (**Figure 7F**). These findings collectively suggest that the inhibition of *SPP1* in HCC-TAMs leads to a transition of macrophages toward a phenotype resembling *C1QA* ^+^ Mφ, a favorable TAM subtype in the context of HCC.

## 4 Discussion

In this study, we conducted a comprehensive analysis of bulk and single-cell RNA-seq data to elucidate key biological processes and associated functional cell types in HCC. Our analysis of bulk RNA-seq data from two independent cohorts including, LIHC and LIRI, highlighted the pivotal role of immunosuppression, with a particular focus on liver macrophages, which emerged as central players in the tumor microenvironment, even surpassing T cells in importance according to our scRNA-seq analysis. By integrating bulk RNA-seq with scRNA-seq analyses, we pinpointed the pivotal role of *SPP1* ^+^ macrophages in modulating TIME in liver cancer and demonstrated the inhibition effects of *SPP1* in HCC-TAMs.

Co-expression network analysis has been widely employed in biomedicine research (67), including HCC (68), to uncover disease mechanisms and associated pathways. However, most co-expression analyses have been conducted on bulk tissue data, which can only reflect gene-gene correlation based on the average expression across multiple cell types within tissues. In contrast, co-expression analysis on single-cell RNA-seq data has been less common due to data sparsity and low dimensionality (69), making it challenging to establish reliable correlations. Although the use of GSOA can sometimes implicitly associate gene sets (e.g., genes in a co-expression cluster) with cell type-specific biological functions, in some cases, such associations can be misleading, as exemplified in our study, where the consensus immunosuppression co-expression cluster indeed showed higher enrichment in macrophages than T cells by scRNA-seq analysis, despite being associated with T cells based on GSOA. Not to mention that most of the GO terms pointed to general biological functions (e.g., ECM) that may exist in various cell types, lacking the link of the co-expression cluster to certain cell types. Our study addressed this challenge by mapping bulk-derived co-expression clusters to certain cell types in scRNA-seq. Apart from the most significant co-expression clusters identified in HCC, i.e., LIHC-5 ∩ LIRI-11 for immunosuppression in macrophages and T cells, and two other consensus clusters for proliferation and metabolism (**Figure 2B**; **Supplementary Figure S1A**), our approaches identified several interesting co-expression clusters associated with specific cell types, including the consensus angiogenesis cluster LIHC-5 ∩ LIRI-11 with LSECs (**Figure 4C**; **Supplementary Figure S1B**), and the tumor-specific ECM cluster LIHC-Diff-11 with HSCs (**Supplementary Figures S2**, **S3B, C**). These interesting clusters and cell types, while deviating from the main scope of this study, have been previously studied for their diverse roles in liver (38). It is expected that further exploration of the key genes within these clusters may uncover novel mechanisms and potential drug targets to modulate the entire tumor microenvironment.

One of the strengths of our study was the successful translation of findings from cell-level analyses based on scRNA-seq to bulk RNA-seq data through *in silico* cell-type deconvolution. This allowed us to estimate the abundance of specific immune cell types in large-scale RNA-seq cohorts and link TIME with patient prognosis. As a recently developed bioinformatic pipeline, many software packages for *in silico* cell-type deconvolution have been developed and benchmarked (70, 71), promoting the wide application of this approach in estimating the TIME (5) in cancer studies. Most of the applications simply deconvoluted the bulk RNA-seq based on pre-trained signatures obtained from data mining in publicly available resources, e.g., the 22 hematopoietic cell types in CIBERSORT (72), which often only result in the estimation of specific cell types that may not perfectly fit the tissue to be analyzed, this study addressed this issue by using the scRNA-seq expression data as the reference to deconvolute bulk RNA-seq from the same tissue/disease. By separately analyzing myeloid cell subsets and T cell subsets, we ensured a precise deconvolution that was less likely to be confounded by overlapping signatures between multiple cell types.

Notably, our findings on the unfavorable role of *SPP1* ^+^ Mφ and the favorable role of *CD8* ^+^ CTLs in HCC prognosis were consistent across two independent RNA-seq cohorts and were supported by *in vitro* validation. The SPP1 – CD44 ligand-receptor pair emerged as a crucial communication axis between *SPP1* ^+^ Mφ and various T cell subsets. Indeed, the significance of the SPP1 – CD44 axis between TAMs and T cells (both CD4^+^ and CD8^+^) has been reported in HCC but not in normal liver tissues (39), showing the disease-specific characteristic of this signaling pathway. A spatial transcriptomic study identified the co-occurrence of the presence of *SPP1* ^+^ Mφ in the HCC tumor area and the exclusion of *CD8* ^+^ T cells from the tumor region, implying the immunosuppressive role of *SPP1* ^+^ Mφ on *CD8* ^+^ T cells (73). Indeed, OPN^high^ (osteopontin, encoded by *SPP1*) facilitates M2-like Mφ polarization, and the reduction of Mφ recruitment and M2 polarization leads to increased *CD8* ^+^ T cell infiltration, which is favorable for anti-PD-L1 immune checkpoint blockade in OPN^high^ HCC (74). In colorectal cancer, the *SPP1* ^+^ Mφ were positively correlated with the tumor-specific *FAP* ^+^ fibroblasts, and the high infiltration of *SPP1* ^+^ Mφ and *FAP* ^+^ fibroblasts was associated with poor anti-PD-L1 therapeutic effects (75), highlighting the important role of SPP1 in cancer development. Similarly, *SPP1* expression was also found to be inversely correlated with *CD8* ^+^ T cells in renal cell carcinoma (76). In addition, studies have suggested the critical role of the SPP1 – CD44 axis between macrophages and glioma cancer cells (77) and between cancer-associated fibroblasts and pancreatic cancer cells (78). Based on *in vitro* experiments, the inhibition of *SPP1* in THP-1 differentiated macrophages co-cultured with the A549 cell line can mitigate lung cancer progression in the cell line model and restore T cell activation (79), showing the potential of targeting SPP1 in various cancers. Moreover, another *in vivo* study has demonstrated the mechanisms of the SPP1 – CD44 axis in colon cancer in a mouse model, that the osteopontin acts as an immune checkpoint to suppress T cell activation and promotes tumor immune evasion in colon carcinoma (80). As reported, the expression of osteopontin is reversely regulated by the transcription factor IRF8 (80), where the TF was significantly enriched in the favorable *C1QA* ^+^ Mφ in our study. Considering the differentiation trajectories from *C1QA* ^+^ Mφ toward two distinct macrophage subsets *SPP1* ^+^ (unfavorable) and *VCAN* ^+^ (favorable) (**Figure 4D**), a potential intervention could be designed to interfere with macrophage differentiation so as to reduce the content of unfavorable and adverse macrophages.

Despite the strengths of our study, as demonstrated by consistent results identified from multiple analyzed datasets, there are limitations. The scRNA-seq discovery cohort, while extensive, included samples from a limited number of patients, providing only a partial reflection of macrophage subpopulations. A larger and more comprehensive scRNA-seq cohort that covers a wider range of macrophage subsets in HCC is currently unavailable. Integrating data from multiple studies to address this limitation would require overcoming challenges related to harmonizing technical and biological batch effects. In addition, future *in vitro* validation would need to demonstrate the effect of Mφ-secreted SPP1 act on T cells via CD44. Nevertheless, the consistency of our findings in two independent cohorts and an *in vitro* experimental validation of the adverse functions of *SPP1* ^+^ macrophage subsets holds promise for advancing immunotherapy in HCC. In this context, novel strategies that modulate macrophage differentiation in conjunction with multitarget therapeutic approaches can be developed.

In conclusion, our study sheds light on the complex interplay between the immune cell types in the liver tumor microenvironment, with a focus on *SPP1* ^+^ macrophages and *CD8* ^+^ CTLs. We highlight the potential of targeting SPP1 in HCC treatment and emphasize the importance of translating insights from single-cell analyses to bulk RNA-seq data to translational clinical applications. These findings contribute to our understanding of HCC immunology and offer new perspectives for novel therapeutic interventions.

## Data Availability

The original contributions presented in the study are included in the article/**Supplementary Material**. Further inquiries can be directed to the corresponding authors. The gene expression data for the TCGA-LIHC and ICGC-LIRI-JP cohorts can be accessed from their respective websites: TCGA-LIHC [https://portal.gdc.cancer.gov/projects/TCGA-LIHC] and ICGC-LIRI-JP [https://dcc.icgc.org/projects/LIRI-JP]. The scRNA-seq discovery and validation data are available on the Gene Expression Omnibus (GEO) under the following accession numbers: GSE166635, GSE140228, and GSE156337. The RNA-seq data for HCC-TAMs can be found on GEO under accession number GSE23066.
