# Supplementary material for: Identification of SPP1 + macrophages as an immune suppressor in hepatocellular carcinoma using single-cell and bulk transcriptomics
Source: Front Immunol. 2024 Dec 3;15:1446453. doi: 10.3389/fimmu.2024.1446453 (PMC11649653; doi:10.3389/fimmu.2024.1446453)
Supplement: Supplementary file 1 [file DataSheet1.docx]

Supplementary Material

# Captions for supplementary tables

Table S1. Differential expression analysis of genes in TCGA-LIHC and ICGC-LIRI-JP cohorts.

Table S2. Clustering results of WGCNA of TCGA-LIHC and ICGC-LIRI-JP cohorts.

Table S3. GSOA of TCGA-LIHC and ICGC-LIRI-JP WGCNA clusters.

Table S4. GSEA of TCGA-LIHC and ICGC-LIRI-JP WGCNA clusters based on gene differential expression.

Table S5. Gene differential expression analysis and markers of five myeloid cell populations and five T cell populations in scRNA-seq data GSE166635, respectively.

Table S6. The relative proportion of myeloid cell subpopulations in liver myeloid cells and T cell subpopulations in liver T cells in TCGA-LIHC and ICGC-LIRI-JP cohorts, analyzed by CIBERSORTx.

Table S7. Results of SCENIC analysis based on scRNA-seq data GSE166635.

Table S8. GSOA of markers of five myeloid cell populations and five T cell populations in scRNA-seq data GSE166635, respectively.

# Supplementary Figures

**
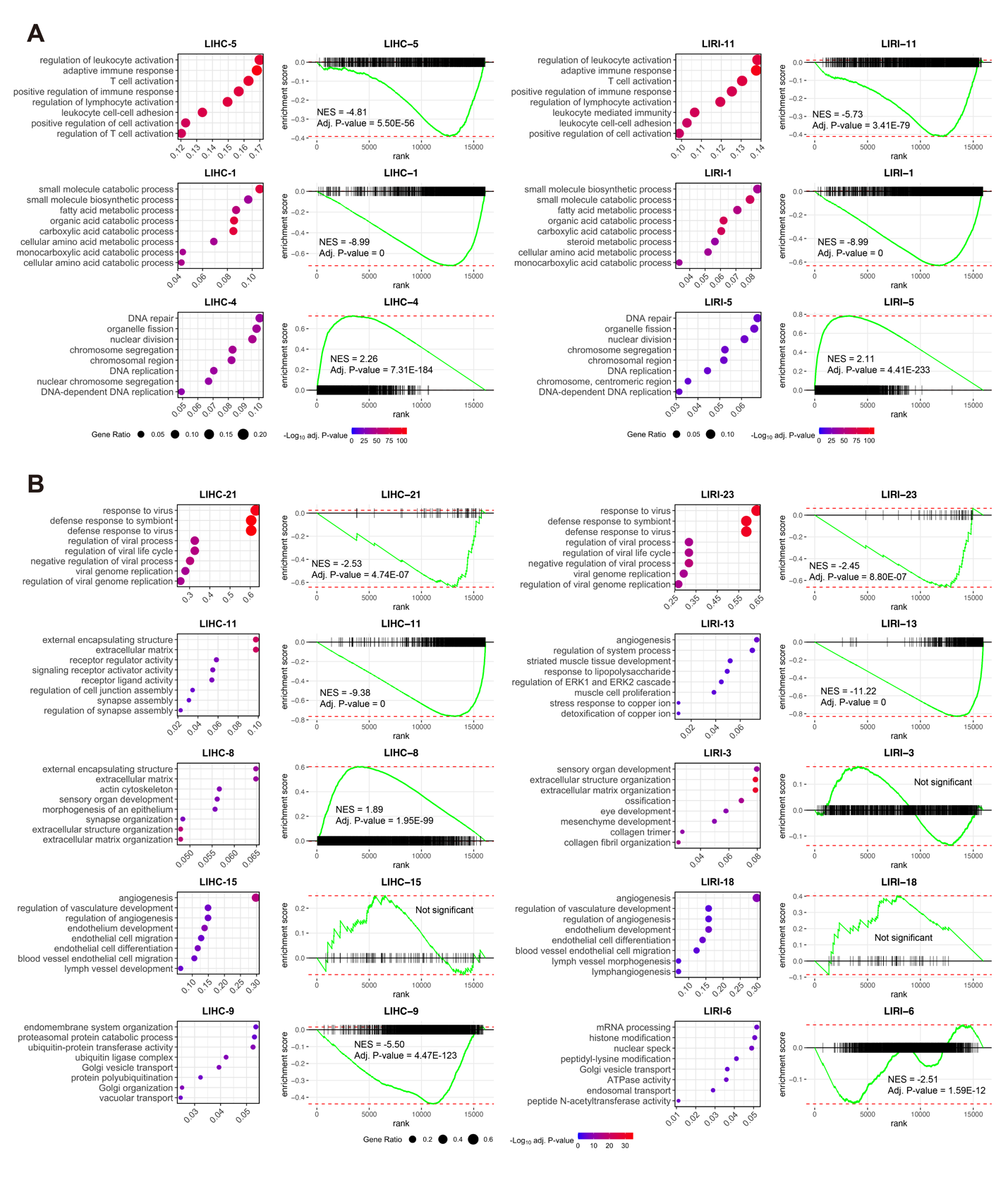
**

**Figure S1. GSOA and GSEA of WGCNA clusters.**

(A) GSOA and GSEA of the WGCNA clusters LIRI-11, LIRI-1, and LIRI-5. For GSEA, genes in the LIRI cohort were sorted based on log_2_ fold change in tumor vs. normal in descending order. For comparison, the corresponding results of the LIHC cohort in Figure 1B are shown aside.

(B) GSOA and GSEA of the matched WGCNA clusters in the LIHC and LIRI cohorts. For GSEA, genes were sorted based on log_2_ fold change in tumor vs. normal in LIHC or LIRI in descending order, respectively.


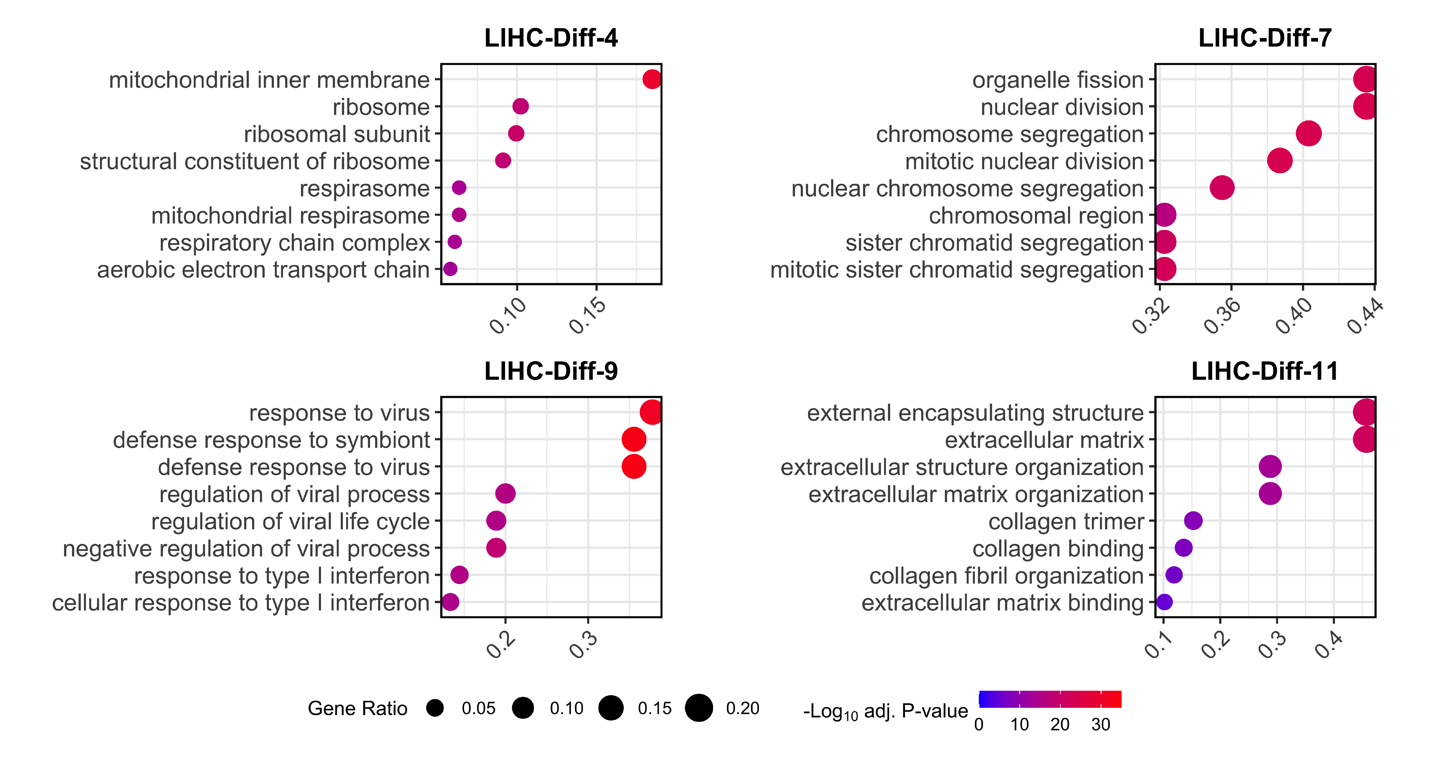


**Figure S2. GSOA and GSEA of differentially co-expressed clusters.**

GSOA of the differentially co-expressed clusters in Figure 1H (without LIHC-Diff-10 and less biologically significant co-expression clusters).


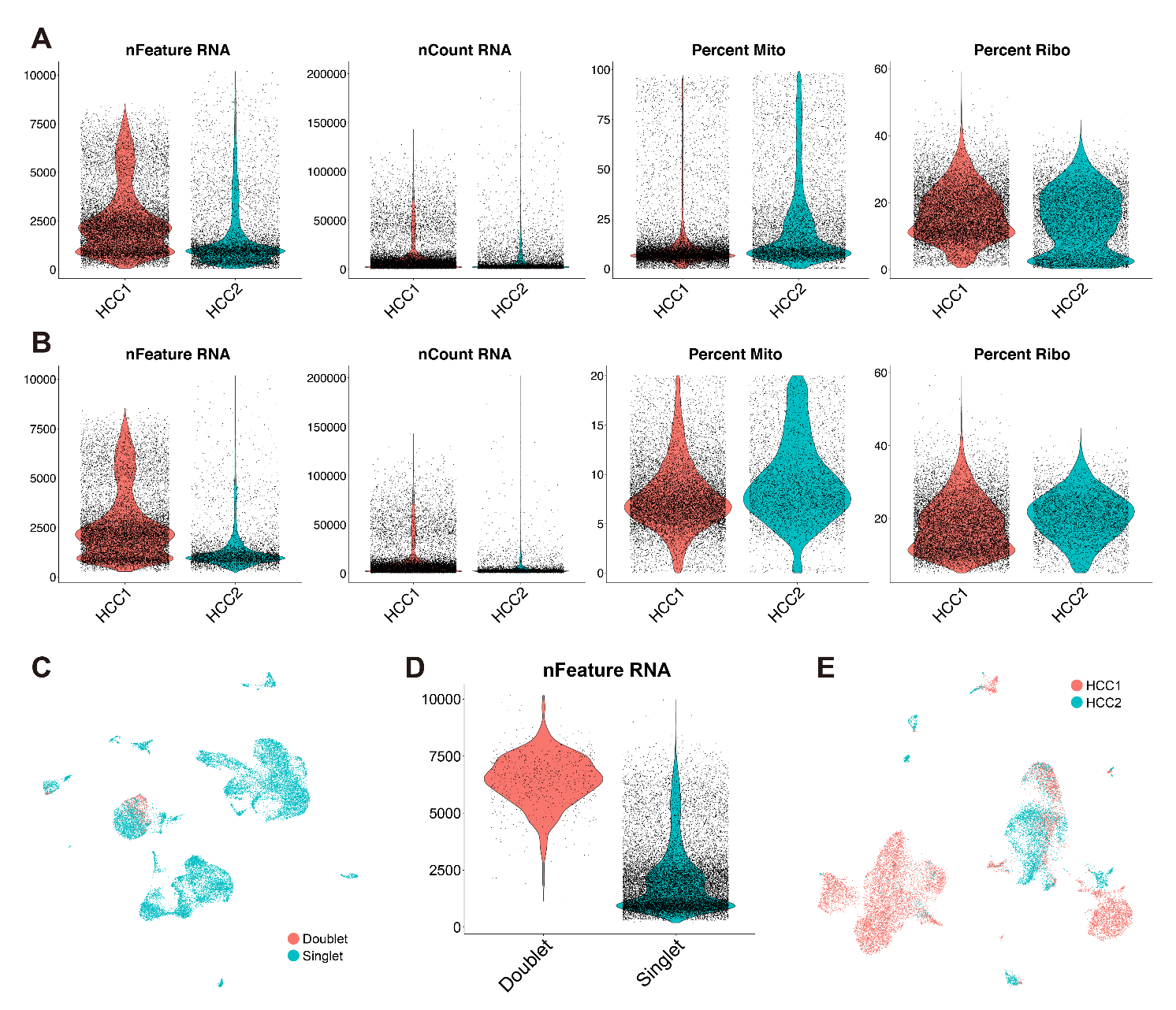


**Figure S3. Preprocessing of liver scRNA-seq data GSE166635.**

(A-B) Violin plots show the number of detected genes (nFeature RNA), the number of counts (nCount RNA), the percentage of mitochondrial reads (Percent Mito), and the percentage of ribosomal reads (Percent Ribo) per cell in the HCC1 and HCC2 samples from the GSE166635 before quality control (A) and after quality control (B).

(C) A two-dimensional UMAP plot shows the detected doublets.

(D) The number of detected genes (nFeature RNA) in the predicted doublets and singlets.

(E) A two-dimensional UMAP plot shows the distribution of the cells from HCC1 and HCC2 samples after quality control.


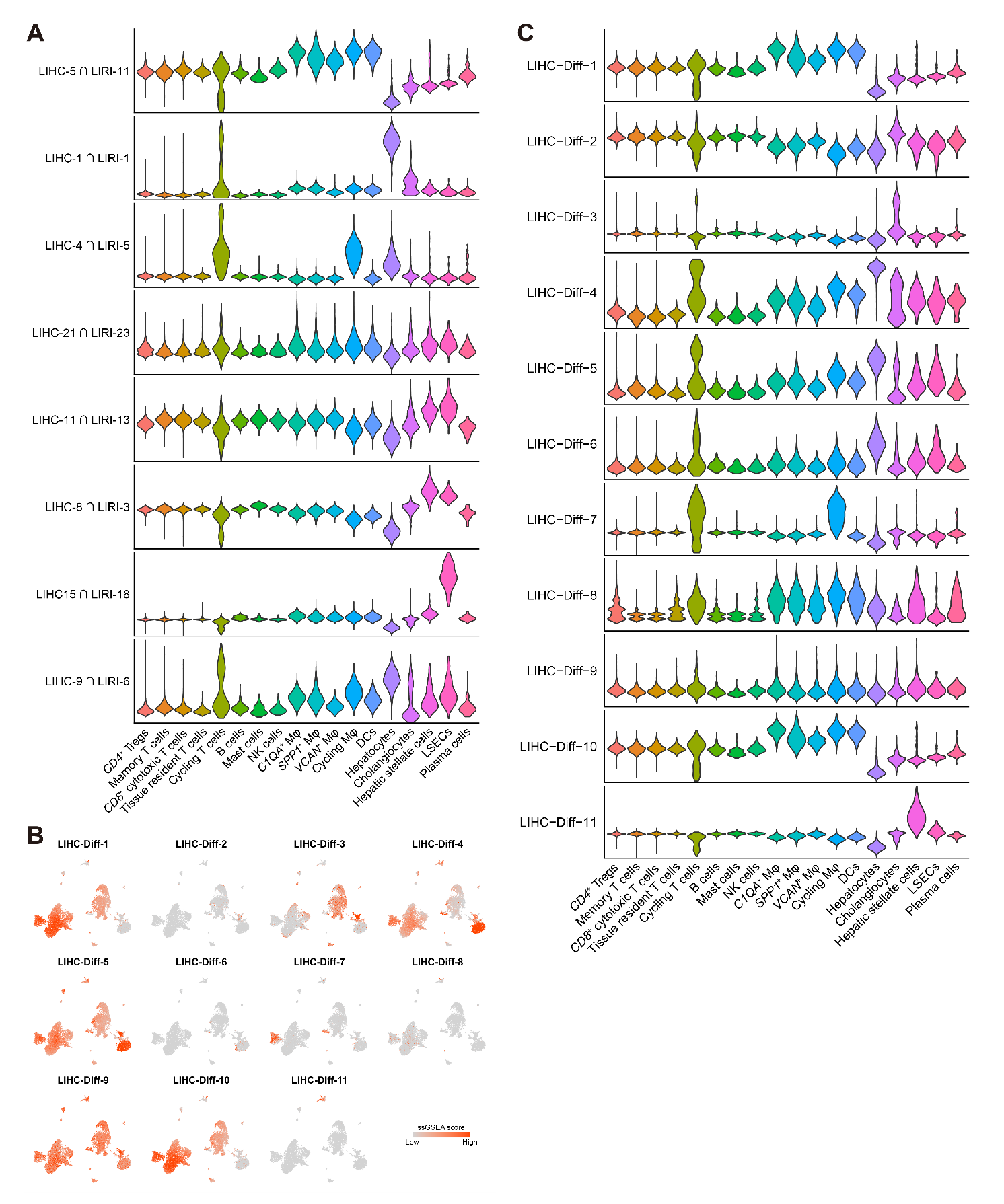


**Figure S4. Enrichment of co-expression clusters in liver scRNA-seq data GSE166635.**

(A) Violin plots show the Enrichment of the intersected genes between LIHC and LIRI consensus co-expression clusters in the scRNA-seq discovery cohort evaluated by ssGSEA.

(B-C) Enrichment of the LIHC differentially co-expressed genes in the scRNA-seq cohort evaluated by ssGSEA, as shown by UMAP plots (B) and violin plots (C).


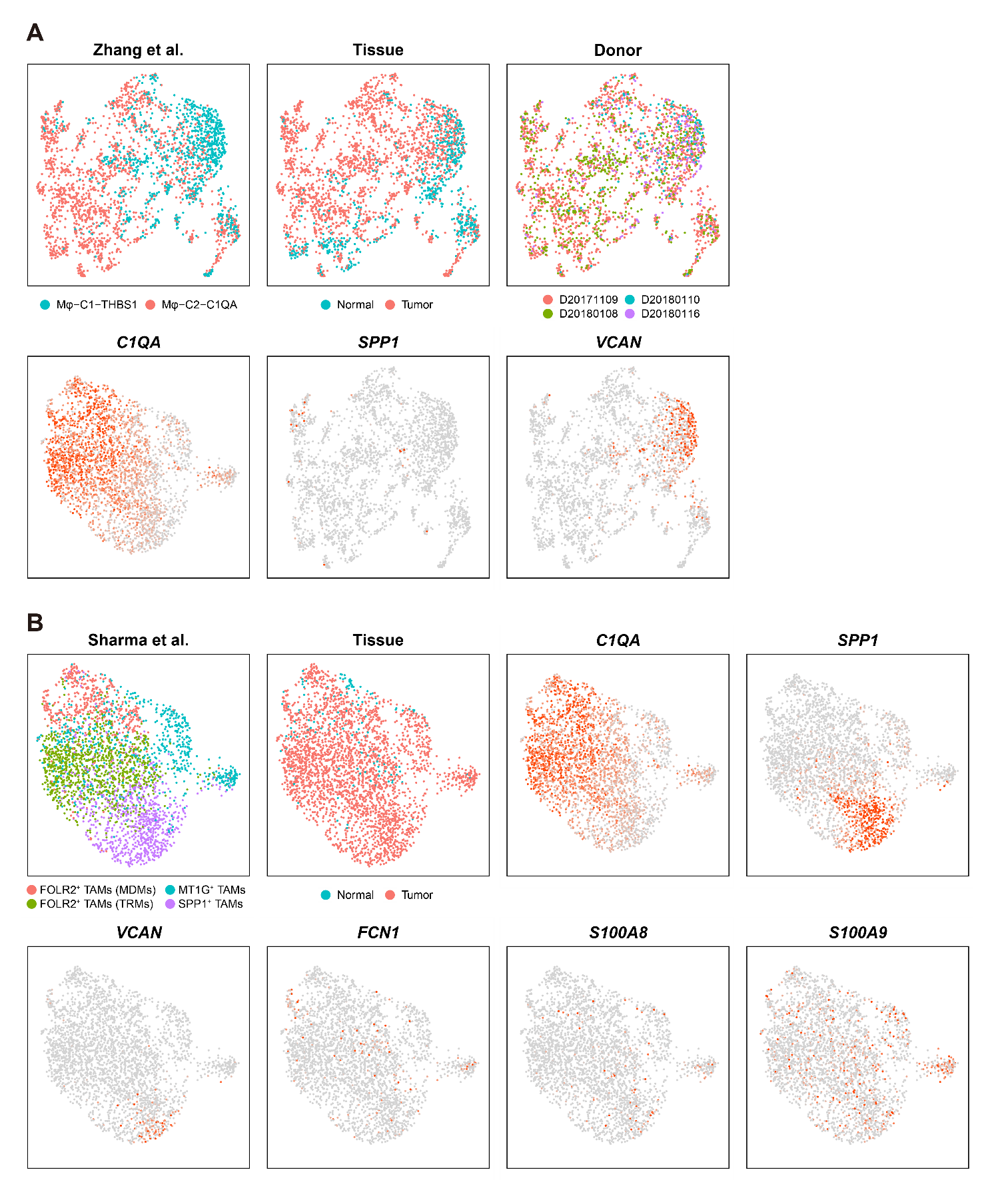


**Figure S5. Validation of the selected markers for liver macrophages in two independent cohorts.**

(A) Validation of the selected markers for liver macrophages in the GSE140228 cohort, related to Figure 3E. Two macrophage subsets Mφ-C1-THBS1 (n = 1,005) and Mφ-C2-C1QA (n = 1,702) in GSE140228 are shown. Cells are visualized in a two-dimensional UMAP plot by cell types, tissue phenotype (normal vs. tumor), or donor origin. Expression of *C1QA*, *SPP1*, and *VCAN* is shown.

(B) Validation of the selected markers for liver macrophages in the GSE156337 cohort, related to Figure 3F. Four macrophage subsets *FOLR2*^+^ TAMs (MDMs; n = 504), *FOLR2*^+^ TAMs (TRMs; n = 1,063), *MT1G*^+^ TAMs (n = 656), and *SPP1*^+^ TAMs (n = 821) in GSE156337 are selected for validation. Cells are visualized in a two-dimensional UMAP plot by cell types or by tissue phenotype (normal vs. tumor). Expression of *C1QA*, *SPP1*, *VCAN*, *FCN1*, *S100A8*, and *S100A9* are shown.


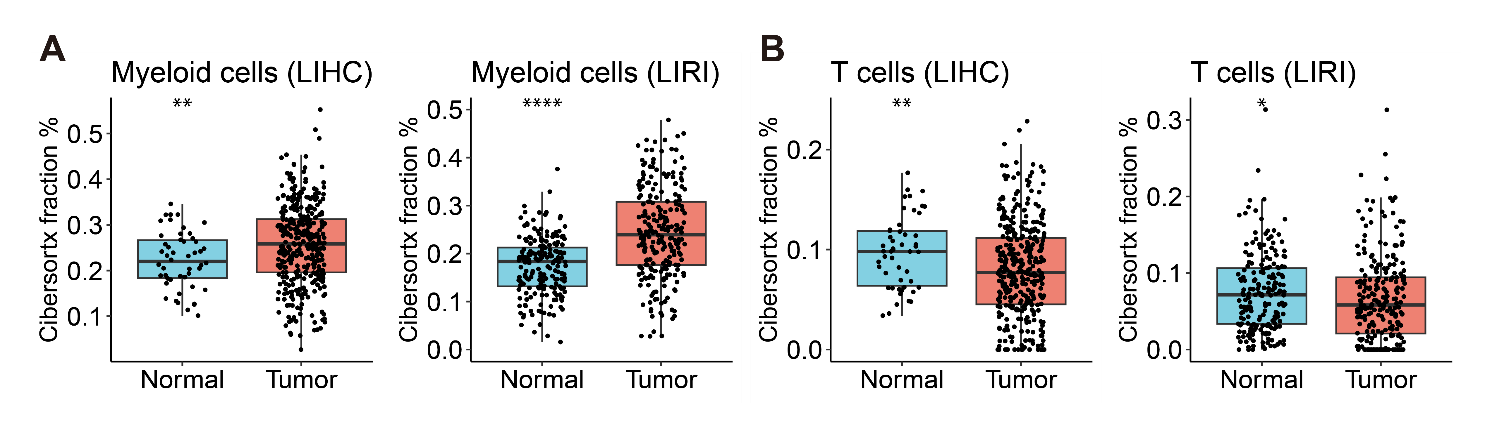


**Figure S6. Fractions of myeloid cells and T cells in LIHC and LIRI cohorts.**

(A-B) The fractions of myeloid cells (A) and T cells (B) in LIHC and LIRI cohorts. The cell type fractions were estimated by CIBERSORTx and were relative to all cell types identified from the discovery scRNA-seq cohort.


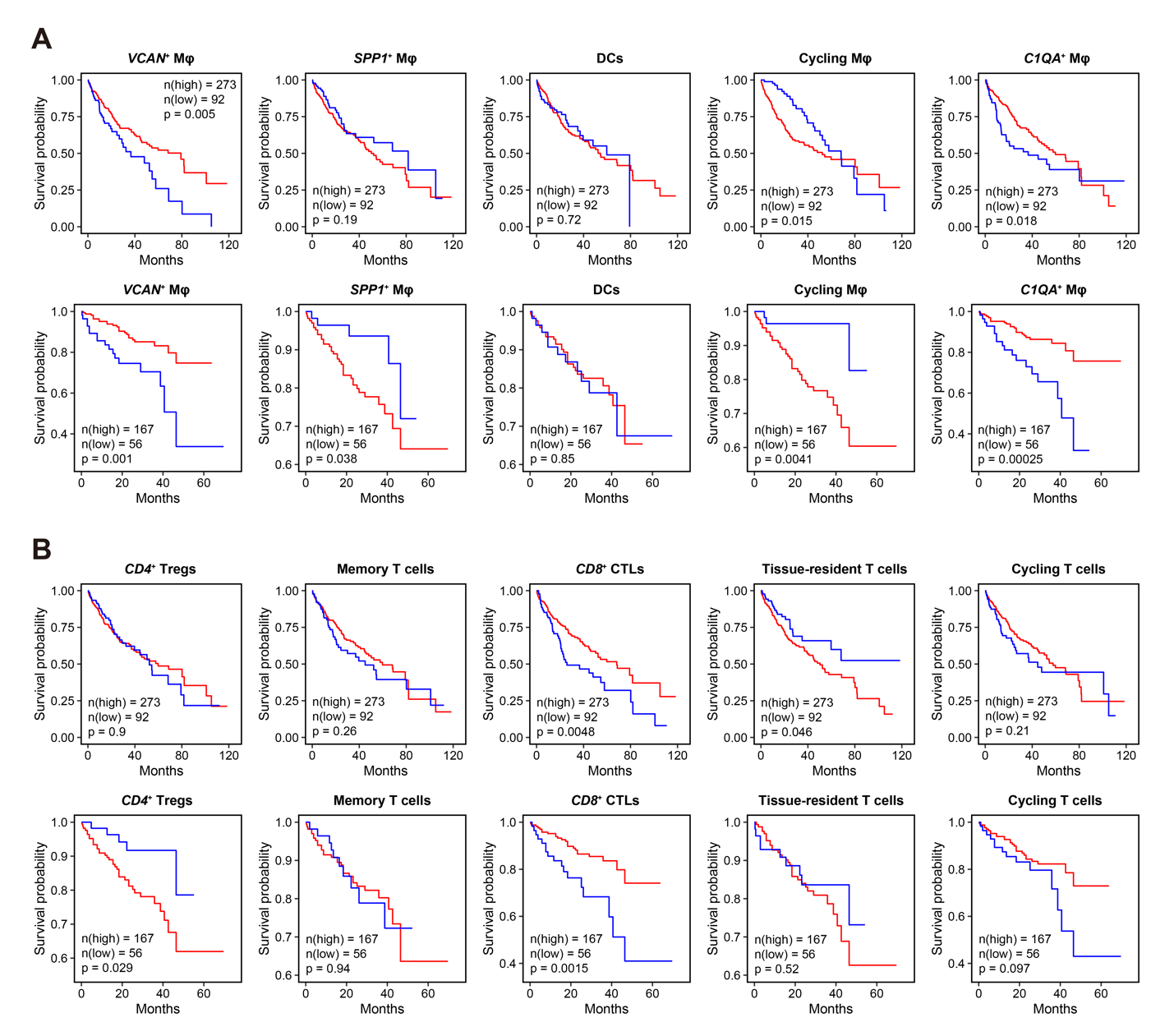


**Figure S7. Kaplan-Meier survival analysis of the immune cell types to patients’ survival.**

(A) Kaplan-Meier survival analysis associates the CIBERSORTx predicted relative fractions of liver macrophages/DCs to patients’ survival based on the LIHC cohort (up) and LIRI cohort (down).

(B) Kaplan-Meier survival analysis associates the CIBERSORTx predicted relative fractions of liver T cell subsets to patients’ survival based on the LIHC cohort (up) and LIRI cohort (down). For (A-B), patients were segregated into high (above the first quartile; red line) and low groups (below the first quartile; blue line) for each cell type based on the CIBERSORTx fractions.


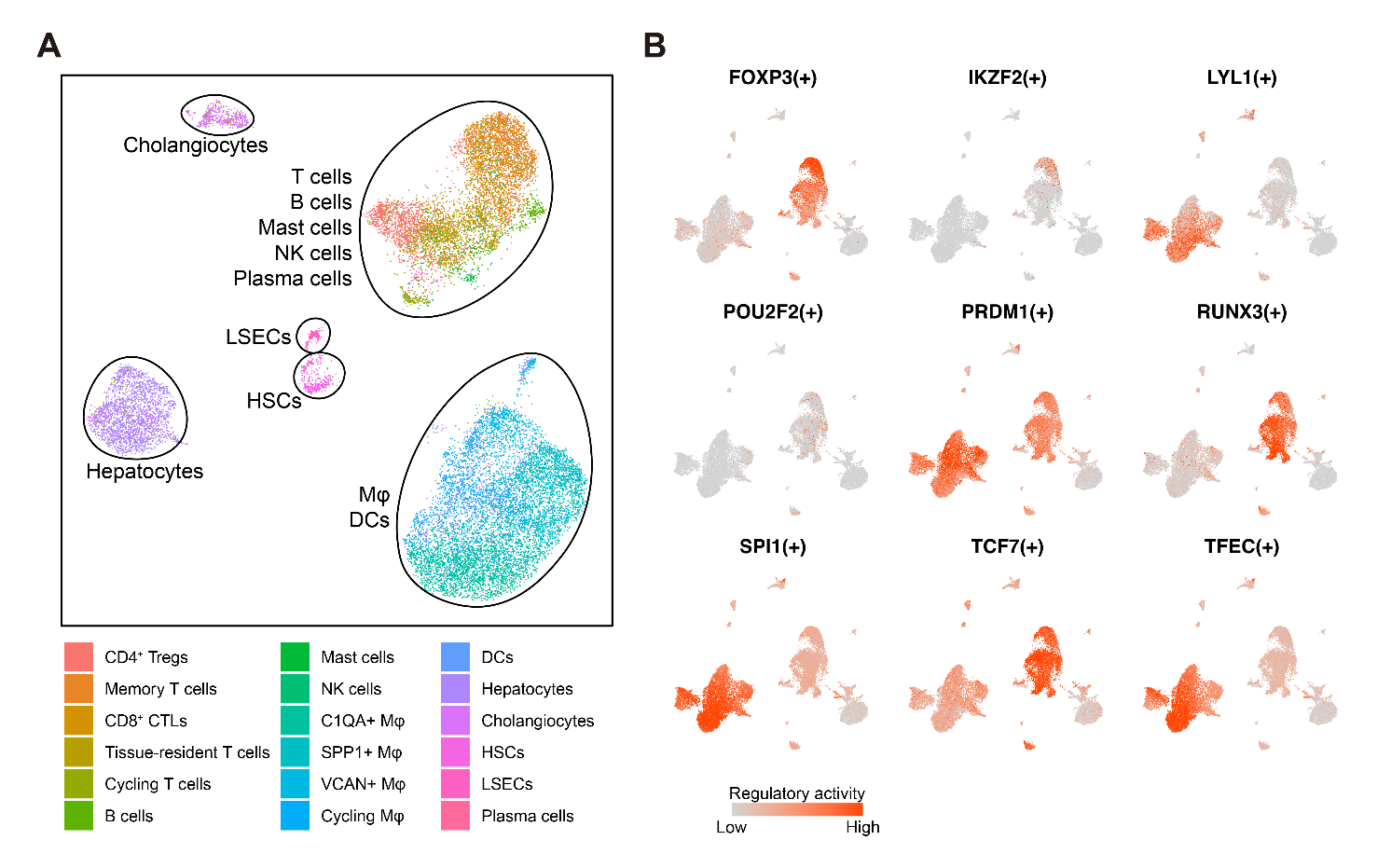


**Figure S8. Single-cell regulatory analysis.**

(A) Two-dimensional UMAP visualization of the HCC cells in the scRNA-seq GSE166635 cohort based on the TF-cell enrichment score.

(B) Enrichment of the TFs regulating the consensus immune co-expression cluster (in Figure 2F) in the scRNA-seq discovery cohort.


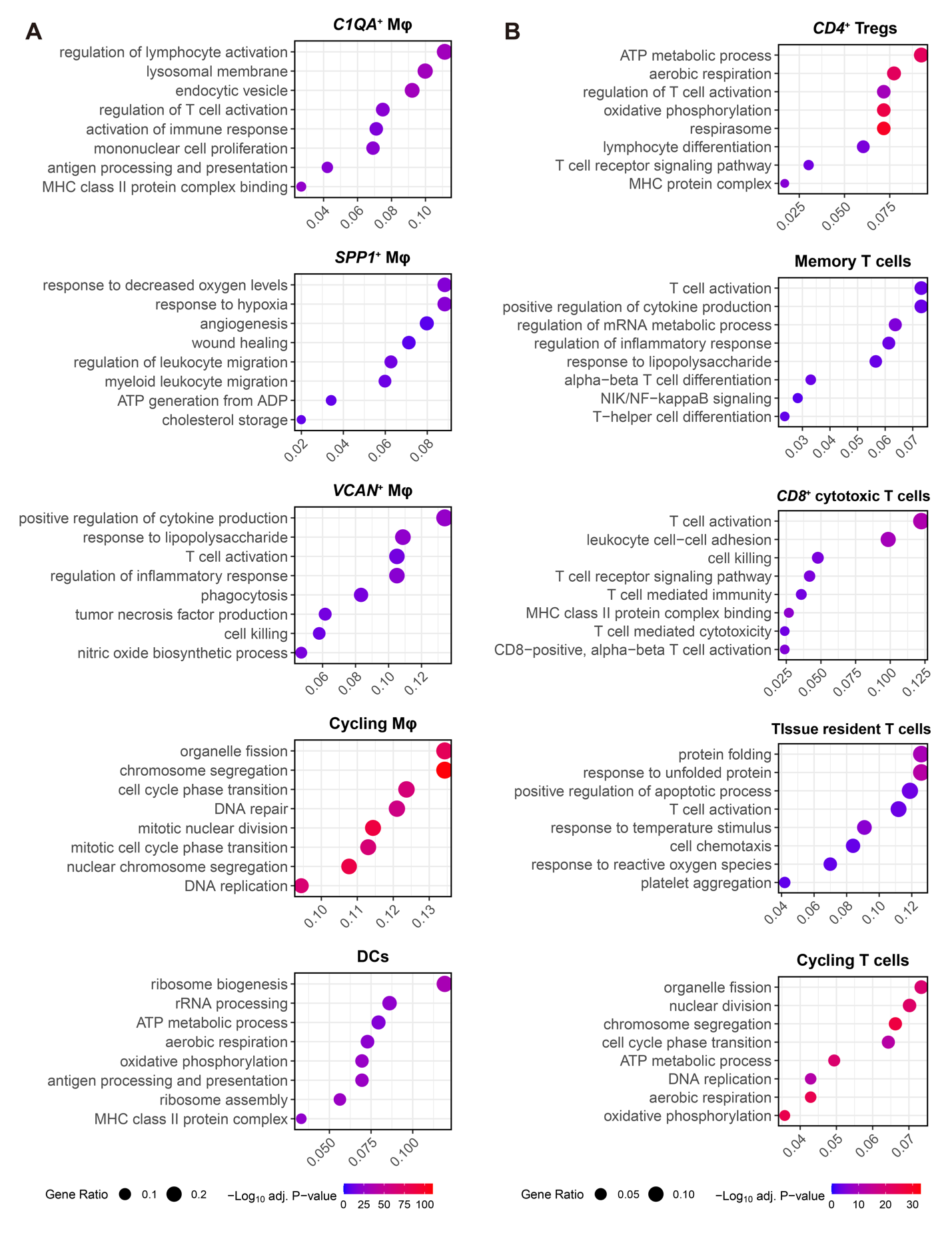


**Figure S9. GSOA of the selected markers for liver immune cells.**

(A) GSOA of the markers for liver macrophages/DCs.

(B) GSOA of the markers for liver T cells. For (A-B), markers were obtained from the scRNA-seq GSE166635 cohort by differential expression analysis.
